# The phenology of the European flat oyster (*Ostrea edulis*) and its environmental drivers

**DOI:** 10.1038/s41598-026-63105-x

**Published:** 2026-07-27

**Authors:** Alexandre Le Moal, Damien Tran, Laura Payton, Yannick Geerebaert, Bernadette Pogoda, Bettina Meyer

**Affiliations:** 1 Institute for Chemistry and Biology of the Marine Environment, Carl von Ossietzky University of Oldenburg, 26111 Oldenburg, Germany; 2 Section Polar Biological Oceanography, Helmholtz Centre for Polar and Marine Research, Alfred Wegener Institute, Am Handelshafen 12, 27570 Bremerhaven, Germany; 3 University Bordeaux, CNRS, Bordeaux INP, EPOC, UMR 5805, Arcachon, F-33120 France; 4 GDR2202 - Lumière & environnement nocturne (LUMEN), UMR 5602 GÉODE, CNRS, University of Toulouse - Jean Jaurès, Toulouse, F-31058 France; 5 Section Shelf Seas Systems Ecology, Alfred Wegener Institute Helmholtz Centre for Polar and Marine Research, Sylt, Bremerhaven, Helgoland, Germany; 6 Helmholtz Institute for Functional Marine Biodiversity at the University of Oldenburg (HIFMB), Im Technologiepark 5, 26129 Oldenburg, Germany

**Keywords:** Bivalve shellfish, Valve behavior, Restoration, Shell growth, Field experiment, Phenology, Ecology, Ecology, Zoology

## Abstract

The European flat oyster (*Ostrea edulis*) is an ecosystem engineer that structures reef habitats and is a major focus of restoration efforts across Europe, yet its biological rhythms in its habitat remain unknown. These rhythms synchronize physiology with environmental cycles, indicating ecological adaptation. Here, we investigated behavioral, physiological, and molecular rhythms in *O. edulis*, as well as their environmental drivers. Using high-resolution valvometry, we monitored valve activity and shell growth over 540-days in the German Bight (Helgoland), along with environmental variables such as photoperiod, temperature, underwater sound, and food availability. Monthly gene expression profiling of clock-related genes was also performed. *O. edulis* displayed coordinated daily, tidal, lunar, semilunar, and annual rhythms, mainly driven by photoperiod and temperature. This integrative chronobiological approach reveals a multiple rhythm system in *O. edulis*, highlighting both adaptive capacity and vulnerability to anthropogenic pressures, and providing key indicators for the conservation and restoration of oyster reef habitats.

**Supplementary Information:**

The online version contains supplementary material available at 10.1038/s41598-026-63105-x.

## Introduction

European flat oyster *Ostrea edulis* (Linnaeus 1758) reef habitats are among the most endangered and degraded marine habitats in Europe^1–3^. Once widespread along the European coast, they have declined dramatically due to a combination of overfishing, habitat destruction, disease, and anthropogenic pressure^2,4,5^. Today, they have almost entirely disappeared from their historical habitats, making them a priority for conservation and restoration. The species and the habitat are listed as threatened and/or declining by the Oslo-Paris-Commission (OSPAR) under the Convention for the Protection and Conservation of the North-East Atlantic^6^, biogenic reefs are protected under EU Flora-Fauna-Habitat Directive (FFH Directive, 92/43/EWG)^7^ and the Marine Strategy Framework Directive (MSFD)^8^ and the habitat type reef (EU code 1170) is to be restored under the EU Nature Restoration Regulation (NRR)^6^.

Restoring these habitats is not only an ecological necessity but also a unique opportunity to restore vital ecosystem functions^9^. *O. edulis* is a filter-feeder bivalve that plays a key role in maintaining water quality, promoting nutrient cycling, and increasing local biodiversity^10,11^. By forming biogenic reefs, these oysters provide shelter and substrate for a variety of marine species, acting as ecosystem engineers^6^. Their presence creates structurally complex environments that can stabilize sediments and buffer coastal erosion^12,13^. Consequently, restoring oyster populations contributes to both biodiversity enhancement^10,12,14^ and climate change resilience^13,15^.

Despite growing interest in restoration efforts, understanding of *O. edulis* biology, particularly in their natural environment, remains limited^16,17^. Gaining insight into their phenology is crucial for identifying factors to strengthen and optimize restoration strategies. However, such knowledge has historically been difficult to obtain, as the marine environment presents numerous technical and logistical challenges for long-term, in situ monitoring. Existing research has primarily focused on aquaculture settings^18^ and the reproductive biology of the species^19,20^. *O. edulis* is a gregarious reef-forming bivalve with a planktonic larval stage followed by permanent settlement on shell substrates preferably conspecific shells, leading to the formation of complex biogenic reef structures^11,21^. Its valve activity, as in other filter-feeding bivalves, regulates feeding, respiration, and reproductive functions and is itself influenced by environmental conditions such as temperature, salinity, and suspended particulate matter^22,23^. Recent studies have now investigated the in situ daily valve activity of *O. edulis* in both field^24^ and laboratory conditions^25^. These studies showed that valve behavior in *O. edulis* responds to environmental conditions and reflects key physiological processes such as feeding and respiration, but they remained limited to short-term or daily-scale investigations, leaving seasonal and long-term phenological rhythms largely unexplored. Thus, a significant knowledge gap still exists regarding how oysters behave and function in their natural, dynamic marine habitats.

Historically, oyster reefs were widespread along European coastal waters, from the Atlantic coast to the Mediterranean Sea, including the Black Sea^2^. One of the most notable examples of this historical abundance was found in the North Sea, covering approximately 20,000 to 25,000 km^2^^19,26^, particularly in the German Bight and around the island of Helgoland^27^. According to an estimate from the Biological Institute of Helgoland (BAH), the Helgoland oyster bed comprised about 1.5 million individuals in 1900, but as in many other regions, the species vanished entirely in the 1940 s, due to overfishing, habitat destruction, and diseases, with no remaining natural populations^5,28,29^. Today, reintroduction and restoration of *O. edulis* in the German Bight contribute to the overarching objectives of ecosystem health, reversal of biodiversity loss and climate resilience^30^. In response, several initiatives have recently emerged at the European level, such as the Native Oyster Restoration Alliance (NORA), which has created a network of scientists, experts, and professionals dedicated to restoring *O. edulis* populations across Europe^31,32^. In Germany, a restoration program has been underway since 2016 to develop and implement sustainable measures to restore European oyster reef habitats in Marine Protected Areas of the German Bight.

To ensure the long-term success of these efforts, it is important to investigate how *O. edulis* interacts with its environment on both biological and temporal levels. Like many organisms, oysters exhibit biological rhythms, that are governed by endogenous molecular clocks^33–36^. These clocks are synchronized with external environmental cues and control a wide range of physiological and behavioral processes, such as feeding, metabolism, and reproduction. In marine environments, organisms face a complex set of cyclical influences due to the overlapping effects of several astronomical cycles^37^, including daily, tidal, lunar, semilunar, and annual cycles. The temporal coordination of biological processes with these cycles is crucial for an organism’s health and fitness^38–40^. In our investigations, we used valve behavior, shell growth, and biological rhythms at gene and behavioral scales as proxies for biological processes and their synchronization with environmental factors to estimate the oysters’ fitness^41–43^. Understanding how *O. edulis* responds to this complex temporal landscape is critical for identifying optimal conditions for growth, reproduction, and survival in natural settings.

This study aims to fill the knowledge gap regarding the phenology of *O. edulis* in its natural environment by investigating its annual valve behavior and shell growth, over an 18-months in situ monitoring period in the German Bight, using high-resolution valvometry under field conditions. This non-invasive technique uses electrodes attached to each shell to continuously record changes in inter-valve distance associated with valve opening amplitude. We monitored the oyster’s valve activity in situ alongside environmental parameters including photoperiod, temperature, water level, underwater sound, chlorophyll *a* concentration, salinity, and turbidity. Additionally, we analysed the monthly gene expression in the gills and labial palps of 13 orthologous genes believed to be involved in the clock machinery, including 9 circadian clock genes (*OeClock*, *OeBmal1*, *OePer1*, *OeTim1*, *OeRev-erb*, *OeRor*, *OeDbt*, *OeShag*, *OeCwo*), a clock-controlled metabolic gene (*OeNampt*), a gene involved in melatonin synthesis (*OeHiomt*), and photoreceptor genes (*OeCry1*, *OeOpn4*). These genes were selected based on their conserved roles in circadian clock function, photoreception, and temporal regulation previously described in vertebrates, insects, and marine invertebrates, including bivalves^34,35,44,45^.

We therefore sought to determine whether *O. edulis* exhibits synchronized behavioral, physiological, and molecular rhythms in its natural environment. We hypothesized that the expression of these rhythms across different temporal scales reflects the species’ good health and could provide a valuable indicator for assessing the suitability of *O. edulis* restoration sites, such as Helgoland. This comprehensive chronobiological approach enables us to assess how behavioral and molecular rhythms align with environmental cycles and how they may be altered in a restored population. This work provides essential knowledge for a deeper understanding of *O. edulis* ecology and supports the development of evidence-based restoration strategies in complex dynamic marine ecosystems.

## Materials and methods

### Experimental model

**Fig. 1:**
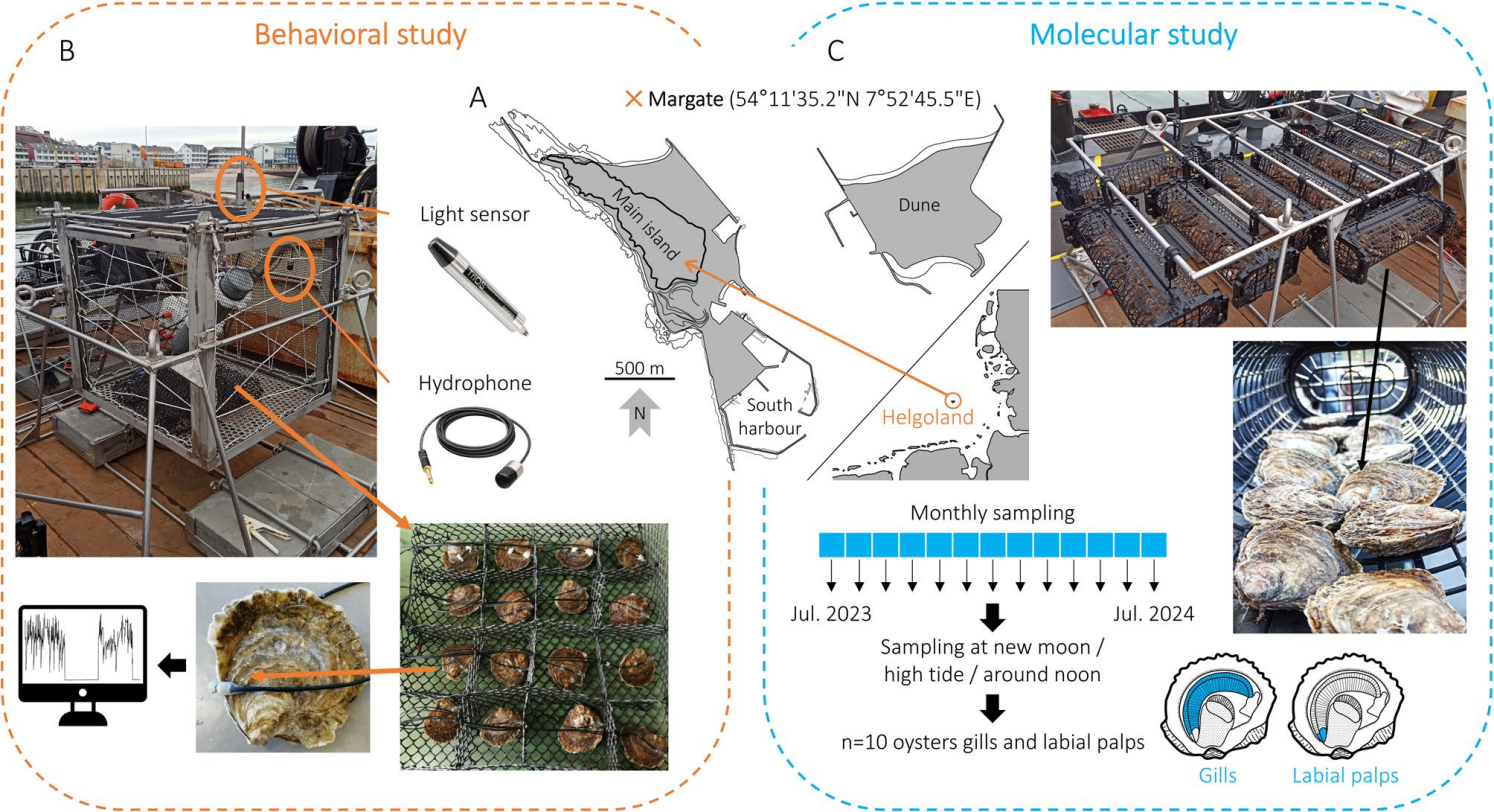
Experimental setup and protocol on Helgoland. (**A**) Study area (island of Helgoland) and its location in the German Bight, North Sea (bottom right corner). The orange cross represents the exact location of the experimental setup at the Margate^47^ (**B**) Experimental setup to investigate the behavioral rhythm of *O. edulis* from March 2023 to August 2024. Picture of the “valvometer lander”, a metallic structure containing a light sensor, a hydrophone, an HFNI valvometer, a basket with 16 European native oysters equipped with electrodes to monitor their valve behavior continuously. (**C**) Experimental setup and protocol of the monthly sampling to investigate the molecular rhythm of *O. edulis* from July 2023 to August 2024. Pictures of the baskets containing oysters for the monthly sampling of oyster gills and labial palps, colored in blue in the diagrams (oyster diagrams: ©2020, Native Oyster Network - UK & Ireland, Native Oyster Restoration Alliance).

All research detailed in this study complied with German law, and the experiment was conducted according to international ethical standards. The experiment was realized in the underwater experimental field “Margate” (54° 11′ 35″ N, 7° 52′ 45″ E) off the island of Helgoland, Germany over a period of 540 days, from 11th March 2023 to 31 st August 2024, involving 156 European native oysters *O. edulis* (approximately 8–10 years old, according to hatchery records). European native oysters were hatchery-reared individuals originating from broodstock collected at Loch Ryan in South West Scotland (54° 59′ 10″ N, 5° 03′ 18″ W) (Rossmore Oysters Ltd.), a sustainably managed source that represents an important broodstock for the Helgoland Oyster Hatchery and German restoration programs^46^. This broodstock was selected in accordance with current recommendations for sustainable oyster restoration and to preserve the genetic diversity of Northeast Atlantic populations used in restoration programs^31^. Prior to field deployment, experimental work was conducted at the Biological Institute Helgoland of the Alfred Wegener Institute Helmholtz Centre for Polar and Marine Research (AWI). Upon arrival, all oysters were accompanied by official veterinary health certificates and had tested negative for *Bonamia* sp. and *Marteilia* sp. Prior to entering the hatchery system, oysters underwent the standard biosecurity protocol, including mechanical cleaning to remove sediment and epibionts, chemical disinfection (freshwater rinse, 0.2% sodium hypochlorite treatment, and final rinse with filtered UV-treated seawater). Following this biosecurity procedure, oysters were acclimated for 3 weeks in outdoor tanks under natural light conditions in a continuous flow-through system using natural seawater from the North Sea, supplemented with a laboratory-cultured microalgae mixture of *Rhodomonas salina*,* Isochrysis galbana* and *Chaetoceros muelleri*. After acclimation, oysters were transferred to the field (Fig. 1). All underwater installations, maintenance checks and sampling of the oyster baskets were conducted by scientific divers from the AWI Centre for Scientific Diving (CSD).

The regulatory framework for research involving living marine organisms is provided by the Nature Reserve Ordinance *Helgoländer Felssockel* and the management plan for the Fauna-Flora-Habitat area DE-1813-391 Helgoland with *Helgoländer Felssockel*.

The study was divided into a behavioral and a molecular part.

### Behavioral part

#### Experimental setup

The behavioral part was conducted on 16 European flat oysters *O. edulis* with a mean ± standard deviation (SD) of 76.9 ± 4.6 mm shell length (range 69–84 mm) and 79.1 ± 4.9 mm shell width (range 69–86 mm) (Biometric measurements of all monitored individuals are provided in supplementary Table S1). The oysters were divided into 16 individual chambers, which were positioned in a basket (Fig. 1B). Each oyster was equipped with a pair of lightweight electrodes connected to a high-frequency non-invasive (HFNI) valvometer (see below). The oyster basket was attached to a lander, which was deployed on 9th March 2023 at “Margate”, at a depth of approximately 10 m, with the help of the AWI Centre for Scientific Diving (CSD) (Fig. 1A). The stainless-steel lander had a cubic structure with a volume of 838 cm³. It was equipped with mesh frames on each side to protect the oysters from predators, without obstructing water flow (Fig. 1B). The mesh frames were replaced and cleaned monthly in parallel with the monthly samplings described below. The metallic structures showed no visible signs of corrosion or degradation throughout the experimental period, suggesting negligible galvanic corrosion effects on the oysters. Several sensors were integrated into the lander structure to measure environmental parameters simultaneously. Water temperature was measured every 10 s (ADT7420 sensor, AnalogDevices) near the valvometer. Underwater sound intensity was recorded in decibels SPL (dB re 1 µPa) using a hydrophone (Aquarian Audio Products (US), H2a) integrated into the HFNI valvometer system. Underwater sound was measured every 10 s across 16 selected low frequencies (10 Hz, 20 Hz, 30 Hz, 40 Hz, 50 Hz, 60 Hz, 70 Hz, 80 Hz, 90 Hz, 100 Hz, 200 Hz, 300 Hz, 400 Hz, 500 Hz, 600 Hz et 700 Hz) that are relevant to bivalve auditory sensitivity^48^. Light irradiance (W/m²) was measured every 10 min in the 320–950 nm range with a radiometer (TRIOS ACC, RAMSES G2-ACC-VIS-300 m), attached to the lander and pointing to the top (Fig. 1B). The resulting irradiance time series was used to identify light-dark transitions and estimate the daily photoperiod, defined here as the duration of the light phase (h/day). The radiometer also recorded water pressure at the sensor level every 10 min, providing continuous data in bars. These pressure values were then converted to water level using the conversion 1 bar = 10 m of depth. In this study, we refer to water level rather than pressure, as the measured variation corresponds primarily to tidal cycles and secondarily to wave swell. Data were transferred daily to the AWI Centre for Scientific Diving (AWI CSD) using the “COSYNA underwater Node unit” connected via a sea cable to the land that provides power and internet connection to the valvometer and the sensors^49^. Hourly average values of temperature and underwater noise levels for each frequencies recorded throughout the experiment are publicly available in PANGAEA^®^ - Data Publisher for Earth & Environmental Science^50^. In parallel to our experiments, chlorophyll *a*, salinity and turbidity were recorded by the AWI-COSYNA Helgoland Underwater Observatory at “Margate” (Helgoland-UWO − 54°11.700’N/7°52.600’E), which is equipped with a standard sensors unit providing real-time measures of environmental parameters^51^^–53^. The environmental parameters monitored in this study were selected because they are known or suspected to influence biological rhythms and physiological processes in bivalves. These include major environmental time cues such as photoperiod^40^^,54^^,55^, temperature^56^^,57^, and tidal cycles^42^, as well as factors affecting feeding activity and physiological performance, including chlorophyll a concentration (used as a proxy for phytoplankton density)^58^, underwater sound^48^^,59^, salinity^60^^,61^, and turbidity^62^.

#### Recording of valve behavior activity

The valve activity of 16 oysters was measured using a custom-built high-frequency non-invasive (HFNI) valvometer, allowing for the remote and continuous recording of their valve activity^63^. It consists of a pair of lightweight (custom-made) electrodes (< 100 mg) designed to minimize disturbance of the oyster’s behavior. The electrodes are glued to each half-shell, and each electrode is linked to the HFNI valvometer by a flexible wire allowing undisturbed oyster valve movement (Fig. 1B). An electromagnetic field is generated successively by each emitting electrode, and the signal received by the corresponding receiving electrode varies with the inter-valve distance, allowing the measurement of each oyster’s valve activity every 1.6 s. Further details are given in Tran et al. 2023^63^ and Le Moal et al. 2023^64^. The daily recordings were processed using Labview 8.0 (National Instrument, Austin, TX, USA).

To analyze the behavioral rhythm, two parameters of valve activity were measured: the valve opening duration (VOD) and the valve opening amplitude (VOA). For each hour, the individual VOD and VOA are measured. VOD is established from an opening threshold (5% of VOA), setting the state of the animal as “open” or “closed”. If the oyster is continuously open for an hour, the percentage of hourly VOD is 100%. On the contrary, if it does not open at all for an hour, the hourly VOD is 0%. Individual VOA of each oyster is reported each hour as a percentage, where 100% indicates that the valves opened at their maximum amplitude during the whole hour and 0% indicates that the valves were closed over the entire hour; in between all the values of amplitudes are possible. Maximum valve opening was defined empirically as the highest opening amplitude recorded for each individual during the monitoring period, with the reference value updated when new maxima were observed. All individual hourly valve behavior data (VOA and VOD) recorded throughout the experiment in Helgoland are publicly available in PANGAEA^®^ - Data Publisher for Earth & Environmental Science^50^.

The mean hourly VOA and VOD are represented as double-plotted actograms and heatmaps generated in Microsoft Excel using conditional formatting, where each line represents two days. In heatmaps, the mean hourly VOA and VOD are represented using a color scale from 0% to 100%, divided into eleven intermediate ranges. A specific category was assigned to 0%, corresponding to complete valve closure (purple), while values > 0% were divided into ten equal ranges. This discretization was chosen to better distinguish fully closed states from minimal valve opening activity. In an actogram, hourly activity levels superior to the daily median are represented by black bars. In contrast, hourly activity levels inferior to the daily median are represented by light grey bars.

#### Measuring of daily shell growth

Individual daily shell growths were measured using the HFNI valvometer. Shell growth in bivalves is driven by the mantle, which deposits successive layers of calcium carbonate on the inner surface of the shell. Therefore, to measure individual daily shell growth, we recorded the minimal distance between electrodes placed on each oyster’s valves when fully closed, over the 540 days of the experiment. The relative slopes of shell growth were calculated as a percentage for the studied periods. Zero per cent and 100% of growth corresponded to the minimal distance in mm between the electrodes at the beginning and the end of the experiment, respectively. Absolute shell growth, in millimetres (mm) was then estimated by measuring the individual shell lengths at the start and end of the experiment. For each oyster, the relative scale (0–100%) was linearly converted to actual shell growth (in mm), where 0% corresponded to the initial shell length and 100% to the final shell length, thus enabling the translation of the distance between electrodes when valves are closed into daily absolute shell length growth.

### Molecular part

For the molecular part of this field experiment, 156 oysters were placed in 13 oyster baskets, with 12 oysters per basket. The 15 L baskets (SEAPA) had dimensions of 600 × 140 × 260 mm and a mesh size of 6 mm. They were attached to a stainless-steel lander, which was deployed at “Margate”, north of Helgoland, on 9th March 2023 (Fig. 1A). From July 2023 to July 2024, oysters were sampled monthly, with a target sample size of 10 individuals per sampling event. Due to natural mortality, the number of oysters collected varied among sampling dates and ranged from 8 to 12 individuals. All sampled oysters were measured prior to molecular analyses and had a mean shell length of 85.6 ± 8.0 mm SD (range 70–110 mm) and a mean shell width of 80.1 ± 9.2 mm SD (range 42–105 mm) (Biometric measurements of all sampled individuals are provided in supplementary Table S2). All sampling was conducted around the 3rd quarter moon (± 3 days), low tide (± 3 h), and around solar noon (± 2 h) to minimise the varying influence of lunar, tidal, and daily rhythms on gene expression. Due to weather conditions, sampling was possible at each time point, except for January and February 2024, during which sampling occurred during the 1 st quarter moon, low tide, and around solar noon.

Underwater, for each sampling event, one oyster basket was detached from the lander and quickly transferred to the boat where the sampled oysters were placed on dry ice. Oysters were euthanized by rapid freezing on dry ice immediately after sampling to minimize transcriptional changes associated with handling stress prior to tissue preservation. Then, back in the lab, each oyster was numbered, measured, and gills and labial palps were immediately dissected (Fig. 1C). Gills and labial palps were selected because they are directly involved in feeding and respiration and have been previously used in bivalves to investigate light-and rhythm-related molecular processes^44^^,65^^,66^. For gills, a tissue sample (0.12 g) was taken and immediately immersed in 800 µl of RNA Later (Invitrogen™ RNAlater™ Stabilization Solution, Fisher Scientific). For labial palps, the four palps were transferred into 1000 µl of RNA Later (Invitrogen™ RNAlater™ Stabilization Solution, Fisher Scientific). Samples were then stored at 4 °C for 24 h before being transferred to −80 °C for long-term preservation before laboratory analysis.

#### RNA extraction and cDNA synthesis

Total RNA was extracted from gills using Trizol (Invitrogen) and a Direct-zol™ RNA MiniPrep (Zymo Research). The quantity and quality of total RNA were assessed by spectrophotometry (OD230, OD260, OD280). RNA reverse transcription was performed using RevertAid H Minus First Strand cDNA Synthesis Kits (Thermo Scientific).

#### mRNA expression analysis by real-time PCR

Primers for clock and clock-associated genes (*OeClock*, *OeBmal1*, *OePer1*,* OeTim1*, *OeRev-erb*, *OeRor*, *OeDbt*, *OeShag*,* OeCwo*, *OeNampt*,* OeHiomt*, *OeCry1*,* OeOpn4*) as well as housekeeping genes (*OeGapdh*,* OeEf1*, *Oe28S*) were designed using the coding sequences (CDS) of these genes, with the assistance of Primer3Plus (https://www.bioinformatics.nl/cgi-bin/primer3plus/primer3plus.cgi). *OeCry2* was not included in this study because it was identified and characterized in *O. edulis* only after the primer panel and molecular analyses had been designed^45^. Forward and reverse sequences for the primers are detailed in supplementary Table S3. Real-time quantitative PCR (qPCR) was performed with the PowerUp™ SYBR™ Green Master Mix kit (Fisher Scientific). The qPCR reactions were conducted under the following conditions: an initial activation step at 95 °C for 2 min to activate the Dual-Lock™ DNA polymerase, followed by 40 cycles of denaturation at 95 °C for 15 s, and annealing and extension at 60 °C for 1 min to amplify the target cDNA. After amplification, melting curves were generated by gradually decreasing the temperature from 95 °C to 60 °C to verify primer specificity. Relative transcript levels of clock and clock-associated genes were determined using the comparative Ct method (2^−ΔCt^) as described by Livak and Schmittgen^67^, where ΔCt is calculated as Ct_(target gene)_ – Ct_(housekeeping gene)_. Following the recommendations of Vandesompele et al. 2002^68^ and the MIQE guidelines^69^, the stability of the reference genes was assessed using the geNorm algorithm, and gene expression was normalized using the geometric mean of the housekeeping genes *OeEf1*, *OeGapdh* and *Oe28S*.

### Data analysis and statistics

#### Mixed models

The influence of eight environmental parameters: photoperiod (daylength), photoperiod direction (increasing or decreasing photoperiod duration), temperature, water level, underwater sound, chlorophyll *a* concentration, salinity, and turbidity, on valve activity (VOA and VOD, individual hourly datasets) and shell growth (individual daily datasets) was investigated using linear mixed-effects models (LMMs). All models included individual identity as a random effect to account for intra-individual variability.

A two-step modelling approach, similar to the method described by Jamil et al. 2012^70^, was applied. This strategy is widely employed in environmental science to investigate complex relationships between species and their environments. In the first step, we fitted global models for each response variable without interaction, including all eight environmental variables as fixed effects:

*VOA or VOD or Growth ~ Photoperiod + Photoperiod direction + Temperature + Water level + Sound + Chlorophyll a + Salinity + Turbidity + (1 | Individual)*.

Underwater sound data were unavailable after 28 February 2024 due to a technical failure; therefore, this variable was not available for the corresponding period and was handled accordingly within the models.

This model (the Single Trait-Environment Relationships model) allowed us to assess the individual effects of each predictor and identify the most influential environmental variables. Prior to model fitting, collinearity among explanatory variables was assessed using pairwise Pearson correlation coefficients. Correlations among predictors were low to moderate (|r| < 0.5), indicating no problematic collinearity among the environmental variables. Then, the three most significant predictors (based on χ² values) were retained for the second modelling step. In the second step, these three variables were used to construct a more focused model, including all two-way interactions:

*VOA or VOD or Growth ~ Var1 + Var2 + Var3 + Var1:Var2 + Var1:Var3 + Var2:Var3 + (1 | Individual)*.

This Multiple Trait-Environment Relationships model allowed us to explore interactions between key environmental factors while reducing model complexity and limiting potential multicollinearity issues associated with the inclusion of the eight predictors and their interactions^70^^,71^.

All analyses were conducted using R (version 4.2.2)^72^ using the packages “lme4”^73^ and “car”^74^ to get the significance of the test. A significance *p*-value of 0.05 was used for all analyses.

#### Chronobiological analysis

For behavioral data, chronobiological analyses were performed using the software Time Series Analysis Serial Cosinor 6.3. Several steps were necessary to validate a significant rhythm. First, to ensure data quality, the absence of random distribution in the dataset is checked using an autocorrelation diagram, and the absence of a stationary phenomenon is assessed through a partial autocorrelation function calculation^75^. Next, the data were examined for periodicity through spectral analyses, using the Lomb and Scargle periodogram^76^. This periodogram establishes a significance threshold (*p* = 0.95). Finally, rhythmicity is confirmed by the Cosinor model, utilizing the period identified by the Lomb and Scargle periodogram^77^. For a given period, the model is expressed as: Y (t) = Acos (πt/τ + φ) + M + ε (t), where A represents the amplitude, φ is the acrophase, τ denotes the period, M is the mesor, and ε is the relative error. Two critical tests verify the validity of the calculated model and the existence of a rhythm: the elliptic test must be rejected, and the probability for the null amplitude hypothesis must be < 0.05. Rhythmicity in the circalunar range (τ = 29.5 ± 3 d) and the circasemilunar range (τ = 14.8 ± 1.5 d) were investigated over the entire 540-day experiment. In contrast, circadian (τ = 24 ± 4 h) and circatidal (τ = 12.4 ± 2 h) rhythms were evaluated over full seasons with 15 individuals: spring (20/03/2023 to 20/06/2023), summer (21/06/2023 to 22/09/2023), autumn (23/09/2023 to 21/12/2023) and winter (22/12/2023 to 19/03/2024). These analyses were conducted for both valve opening amplitude (VOA) and valve opening duration (VOD) at the individual level (*n* = 15 individuals).

Rhythmic analysis of circannual rhythm in valve activity (VOA and VOD) and clock genes expression was carried out using the R package ‘RAIN’^72,78^. RAIN was specifically designed to detect rhythms in biological datasets, regardless of waveform, employing a non-parametric approach^78^. Analyses were performed on monthly mean values of valve activity and on gene expression data obtained from the monthly sampling. For both datasets, the presence of a significant annual rhythm was tested by specifying a period of 12 months. Rhythmicity was considered significant for a corrected *p*-value less than or equal to 0.05.

#### Temporal segmentation for biological rhythm analysis

To assess variations in valve activity (hourly VOA and VOD) across different biological rhythms, the dataset was segmented according to specific geophysical cycles studied during the chronobiological analysis. For the analysis of annual rhythms, data were divided by season: spring (11/03/2023 to 20/06/2023 and 20/03/2024 to 19/06/2024), summer (21/06/2023 to 22/09/2023 and 20/06/2024 to 31/08/2024), autumn (23/09/2023 to 21/12/2023), and winter (22/12/2023 to 19/03/2024). For lunar rhythms, VOA and VOD values were averaged over a 3-day window centered on each main lunar phase (new moon, first quarter, full moon, and third quarter), with lunar dates retrieved from the website www.timeanddate.com. For the semilunar rhythm (neap-spring tidal cycle), data were averaged over 3-day periods centered on key tidal coefficient phases (high, decreasing, low, and increasing coefficients), with tidal coefficients from Helgoland obtained from the website https://mareespeche.com. For daily rhythms, VOA and VOD were averaged separately for daytime and nighttime periods, defined according to theoretical sunrise and sunset times for each season used in the chronobiological analysis (retrieved from the website www.timeanddate.com). Lastly, for tidal rhythms, for each season used in the chronobiological analysis, data were categorized into four 6-hour phases based on in situ pressure recordings: high tide (6 h of the highest water level), low tide (6 h of the lowest water level), ebb (decreasing water level), and flow (increasing water level). This segmentation allowed for targeted analysis of valve activity aligned with relevant chronobiological cycles.

#### Descriptive statistics

For each temporal segmentation, including annual, lunar, semilunar, daily, and tidal rhythms, differences in VOA and VOD values across the designated phases were tested using the non-parametric Kruskal-Wallis test. This test was chosen because it does not assume normality and is suitable for comparing multiple independent groups with potentially non-Gaussian distributions. When the Kruskal-Wallis test revealed significant differences, pairwise comparisons were conducted using Dunn’s post hoc test, which accounts for multiple comparisons while maintaining robustness for non-parametric data. All analyses were conducted using R (version 4.2.2)^72^ and a significance *p*-value of 0.05 was applied for all analyses.

## Results

### *Ostrea edulis* valve activity

**Fig. 2:**
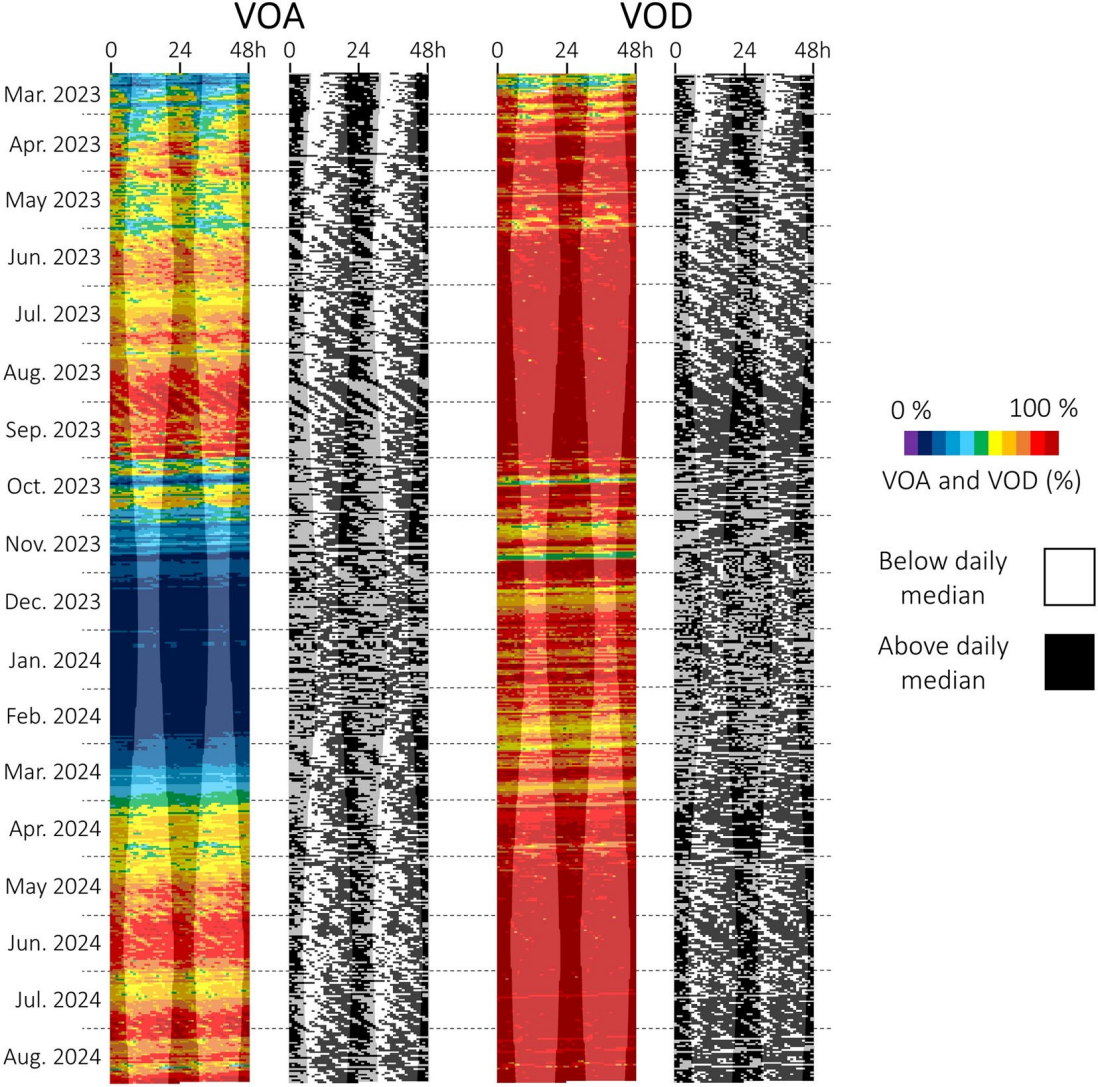
*Ostrea* *edulis* hourly valve activity in the field over the 540 experimental days. Double-plotted heatmaps and associated actograms of mean valve opening amplitude (VOA, %, left panel) and valve opening duration (VOD, %, right panel) from 11th March 2023 to 31 st August 2024 in the island of Helgoland, Germany (*n* = 8–15; 15 oysters until March 2024, 14 until June 2024, and 8 thereafter due to progressive technical issues). Photoperiod is represented in the heatmaps and actograms in transparent shading. In heatmaps, hourly VOA and VOD are shown using a 0–100% color scale, with 0% (complete closure) displayed as a distinct category (purple) and values > 0% grouped into 10 equal intervals.

We continuously monitored the valve activity of *Ostrea edulis* over 540 days in the field. Figure 2 shows the temporal dynamics of valve activity, presented as double-plotted heatmaps and actograms for both VOA (left panel) and VOD (right panel). Hourly mean values are overlaid with photoperiod transparency to aid in temporal interpretation. The corresponding daily valve activity of each individual oyster is presented in Supplementary Fig. S1. One oyster died one day after deployment; the remaining 15 oysters survived the entire experiment (540 days). However, due to technical issues, valve activity was recorded for 15 oysters until March 2024, then for 14 oysters until June 2024, and finally for 8 oysters until the end of the experiment. Despite this progressive reduction in sample size due to technical signal loss, the high-frequency continuous recordings yielded numerous repeated measurements per individual, helping maintain robust estimates of valve activity dynamics throughout the monitoring period.

The heatmaps reveal a clear annual pattern of VOA, with values gradually increasing from early spring (March 2023) to peak in summer (e.g., with mean monthly VOA values reaching 77 ± 2% in August and 81 ± 1% in September 2023), then declining steadily from the start of autumn to late autumn (November 2024), and finally reaching a minimum in winter (as low as 7 ± 1% of monthly VOA in January). After winter 2024, the valve opening increases again until summer, gradually rising from VOA from 40 ± 2% in March to 83 ± 1% in June, following trends similar to those seen in spring and summer 2023 with peak values reached around late July/early August 2024. VOD showed a much less pronounced seasonal pattern. VOD remains nearly maximal throughout the year (88 ± 14%), except during autumn and winter, when it drops to 40 ± 3% and to 53 ± 1% of mean daily VOD, respectively. The VOA and VOD actograms reveal underlying rhythmic patterns in valve activity at the daily and tidal scales that vary seasonally. Clear patterns are visible from March 2023 to mid-November 2023, and again from early March 2024 to the end of the experiment, while a more irregular blurred phase is observed in between, corresponding to the winter period. During periods of pronounced rhythmicity, a consistent diel pattern is evident, often accompanied by an underlying tidal signal that occasionally becomes more prominent, particularly noticeable in early June and late August 2023 (see representative examples in Supplementary Fig. S2).

### Environmental parameters

To understand the environmental factors affecting the biological rhythms of *Ostrea edulis* in the field, we monitored seven abiotic parameters over the 540-day experiment: temperature, photoperiod, water level, underwater sound, chlorophyll *a* concentration, salinity, and turbidity. Collectively, these environmental measurements offer a comprehensive framework for assessing the natural entrainment of biological rhythms and physiological processes in *O. edulis* under natural conditions.

**Fig. 3:**
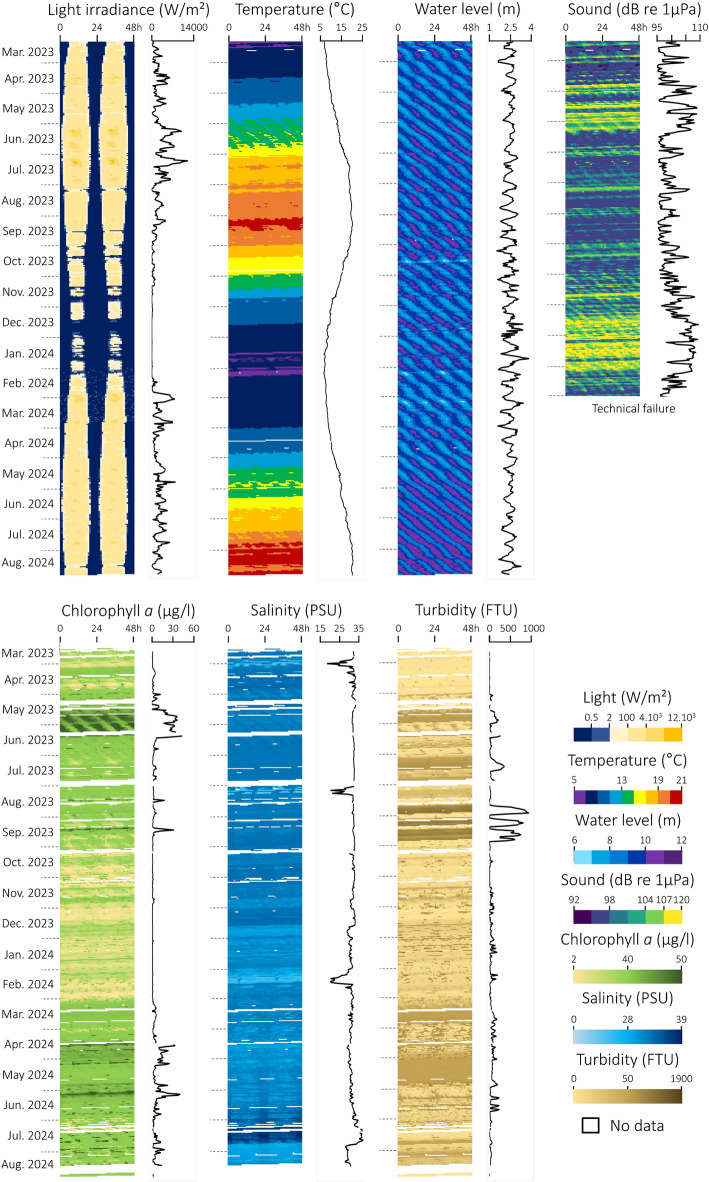
Time series of environmental parameters monitored over the 540 experimental days. Time series of light irradiance (W/m²), temperature (°C), water level variation (m), underwater sound (dB re 1µPa), chlorophyll *a* concentration (µg/l), salinity (PSU) and turbidity (FTU) monitored from 11th of March 2023 to 31 st of August 2024 in the island of Helgoland, Germany. For each parameter, a double-plotted heatmap of hourly means is shown on the left, and a line chart of daily means on the right. For the light, the line chart shows the daily maximum value. For the water level, the line chart illustrates the difference between the daily maximum and minimum values. The underwater sound dataset ends on 28/02/2024 due to a technical failure. White cells represent missing data.

Photoperiod followed the solar cycle, ranging from 7.5 h of daylight at the winter solstice to 17.5 h at the summer solstice, with maximum measured light irradiance reaching 11,979 W/m² at the oyster’s level in July 2023. Temperature exhibited a clear seasonal pattern, gradually rising from winter to summer, reaching 20.7 °C in August 2024. The lowest temperature recorded was 6.42 °C in February 2024. Seasonal averages for 2023/2024 were 11.2 ± 0.1 °C in spring, 18.8 ± 0.1 °C in summer, 14.2 ± 0.1 °C in autumn, and 7.7 ± 0.1 °C in winter. The water level above the oysters fluctuated regularly between 6 and 12 m, driven by tidal cycles and wave swell, with a daily mean oscillation of 2.5 ± 0.1 m. Underwater sound levels exhibited episodic peaks, particularly between mid-May and mid-June 2023 and from December 2023 to January 2024, with intensities ranging from 107 to 120 dB re 1 µPa. Chlorophyll *a* concentration had notable spring peaks, with daily maxima of 43 µg/L on 13 June 2023 and 40 µg/L on 9 June 2024. Seasonal averages were highest in spring (9.5 ± 0.2 µg/L), and much lower in autumn (1.1 ± 0.1 µg/L) and winter (1.0 ± 0.1 µg/L). Salinity remained relatively constant at around 31.4 ± 0.1 PSU, with lower values during rainy winter periods and higher levels in summer (e.g. July 2024). Turbidity exhibited both seasonal and episodic variability, with notable increases in August-September 2023 and May 2024. Average seasonal values were highest in summer (140.1 ± 4.0 FTU) and spring (60.7 ± 1.4 FTU), and lowest in autumn (49.5 ± 2.9 FTU) and winter (42.2 ± 2.5 FTU) (Fig. 3).

### The influence of environmental parameters on *Ostrea edulis* valve activity

Linear mixed-effects models were used to assess the influence of environmental factors on hourly VOA et VOD. The outputs of the Simple Trait-Environment Relationships and Multiple Trait-Environment Relationships models are summarized in Table 1. In the first VOA model, all parameters demonstrated significant effects. Photoperiod had the most influential impact (χ² = 22010), followed by temperature (χ² = 11702) and sound (χ² = 6310). The effects of salinity (χ² = 948), chlorophyll *a* (χ² = 495), and turbidity (χ² = 437) were also significant, while water level effects were marginal (χ² = 21). Positive effects were observed for photoperiod (β = 4.10), temperature (β = 3.45), and water level (β = 3.26), indicating that an increase in these variables is associated with higher VOA; conversely, decreasing photoperiod (β = −12.43) and sound (β = −1.26) had negative effects. In the second VOA model, which focused on temperature, photoperiod, and sound, all three environmental factors were significant, both individually and in interaction. The interaction between temperature and photoperiod had the most decisive influence (χ² = 7788), followed by temperature (χ² = 6087), and the interaction between temperature and sound (χ² = 3366), highlighting a pronounced synergistic effect between temperature and photoperiod or sound, which overshadows the independent influence of photoperiod. For VOD, the first model without interaction revealed significant effects for all parameters except salinity (*p* = 0.081). Temperature (χ² = 802), photoperiod (χ² = 573), and chlorophyll *a* (χ² = 215) were the strongest predictors. Photoperiod direction (χ² = 186), turbidity (χ² = 144), sound (χ² = 104), and water level (χ² = 17) had lower, yet significant effects. The second VOD model, incorporating temperature, photoperiod, and chlorophyll *a*, indicated, that similar to VOA, the predominant effect was a synergic interaction between temperature and photoperiod (χ² = 717), followed by the individual effects of temperature (χ² = 338) and photoperiod (χ² = 157), as well as interactions between chlorophyll *a* and temperature (χ² = 27) and chlorophyll *a* and photoperiod (χ² = 28).

**Table 1:** Results from linear mixed models analysing the effect of environmental parameters on *Ostrea edulis* valve activity (VOA and VOD). Outputs from linear mixed models considering the influence of various fixed factors (photoperiod, photoperiod direction, water temperature, water level, underwater sound, chlorophyll *a* concentration, salinity, and turbidity) on the individuals’ VOA and VOD over the study period (540 days, i.e. 12960 h). A second model examines the influence of the three most significant predictors from the first model (based on χ²) without interaction, as well as all two-way interactions, on individuals’ VOA and VOD over the study period. Significant *p*-values (*p* < 0.05) are indicated in bold.

VOA					VOD				
Single Trait-Environment Relationships model									
VOA ~ Photop. + Photop. dir. + Temp. + Water level + Sound + Chl. *a* + Salinity + Turbidity + (1 | Ind)					VOD ~ Photop. + Photop. dir. + Temp. + Water level + Sound + Chl. *a* + Salinity + Turbidity + (1 | Ind)				
Parameters	*β*	*SE*	*χ2*	*p*	Parameters	*β*	*SE*	*χ2*	*p*
Photop.	4.10	0.03	22,010	**< 0.001**	Photop.	1.01	0.04	573	**< 0.001**
Photop. dir.	−12.43	0.26	2362	**< 0.001**	Photop. dir.	−5.34	0.39	186	**< 0.001**
Temp.	3.45	0.03	11,702	**< 0.001**	Temp.	1.38	0.05	802	**< 0.001**
Water level	3.26	0.72	21	**< 0.001**	Water level	−4.47	1.10	17	**< 0.001**
Sound	−1.26	0.02	6310	**< 0.001**	Sound	−0.25	0.02	104	**< 0.001**
Chl. *a*	−0.18	0.01	495	**< 0.001**	Chl. *a*	−0.18	0.01	215	**< 0.001**
Salinity	0.66	0.02	948	**< 0.001**	Salinity	−0.06	0.03	3	0.081
Turbidity	0.01	0.00	437	**< 0.001**	Turbidity	0.01	0.00	144	**< 0.001**

Multiple Trait-Environment Relationships model									
VOA ~ Photop. + Temp. + Sound + Temp.:Photop. + Temp.:Sound + Photop.:Sound + (1 | Ind)					VOD ~ Photop. + Temp. + Chl. *a* + Temp.: Photop. + Temp.:Chl. *a* + Photop.:Chl. *a* + (1 | Ind)				
Photop.	12.87	0.53	600	**< 0.001**	Photop.	−1.42	0.11	157	**< 0.001**
Temp.	31.89	0.41	6087	**< 0.001**	Temp.	−1.97	0.11	338	**< 0.001**
Sound	1.88	0.06	960	**< 0.001**	Chl. *a*	0.27	0.20	2	0.185
Temp. x Photop.	−0.54	0.01	7788	**< 0.001**	Temp. x Photop.	0.23	0.01	717	**< 0.001**
Temp. x Sound	−0.23	0.00	3366	**< 0.001**	Temp. x Chl. *a*	0.03	0.01	27	**< 0.001**
Photop. x Sound	−0.01	0.01	5	**0.021**	Photop. x Chl. *a*	−0.05	0.01	28	**< 0.001**

*VOA* valve opening amplitude; *VOD* valve opening duration; Photop.: photoperiod (daylength); Photop. dir.: photoperiod direction (increasing or decreasing daylength); Temp.: water temperature; Chl. *a*: chlorophyll *a* concentration. Significant values are in bold.

### *Ostrea edulis* shell growth

**Fig. 4:**
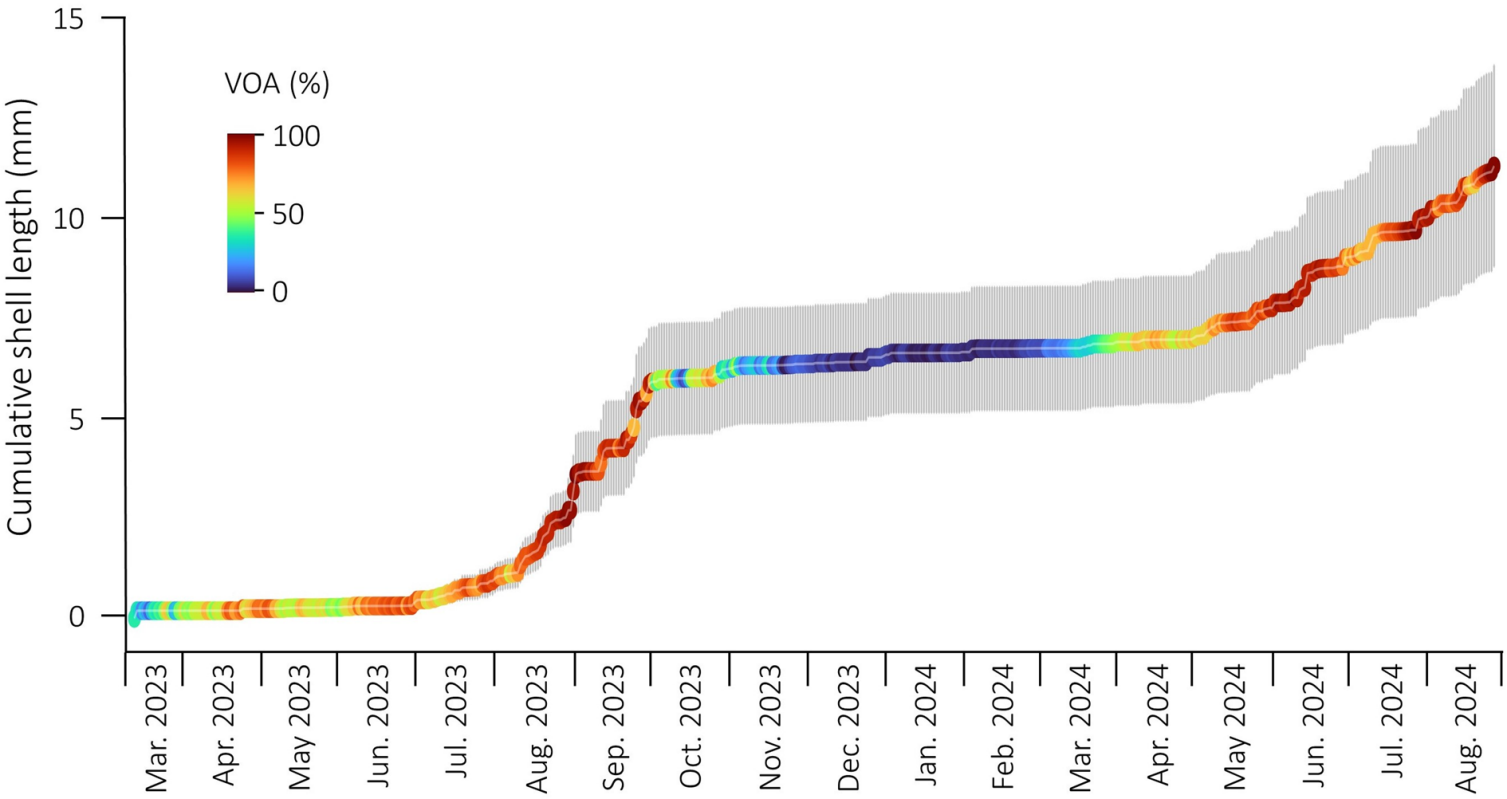
Daily shell growth of *Ostrea edulis* in the field over 540 days of experimentation. Line chart showing the cumulative shell growth of all individuals (*n* = 8–15; 15 oysters until March 2024, 14 until June 2024, and 8 thereafter due to progressive technical issues) from 11th March 2023 to 31 st August 2024 on the island of Helgoland, Germany. The curve is colored by the daily mean valve opening amplitude (VOA, %) to facilitate visual comparison between seasonal patterns of valve activity and shell growth. Error bars represent the standard error (SE).

Shell growth was monitored daily throughout the 540-day experiment. Figure 4 displays the mean (± SE) cumulative shell growth curves. For visualization purposes, the curve is color-coded according to the mean daily VOA, which showed a stronger association with shell growth than VOD and therefore facilitated visualization of their seasonal relationship. The 2023 growing period peaked in summer, then declined in autumn and winter, with a renewed increase starting in spring 2024, particularly in the summer of 2024. The reduced growth observed during spring 2023 reflects interannual variability in the timing of the growth season rather than a lack of growth. While growth onset occurred later in 2023 than in 2024, both years exhibited a similar overall seasonal pattern and a comparable total shell increment. This shift is likely driven by interannual differences in environmental conditions (e.g. temperature, food availability, and light regime) rather than acclimatization, as oysters were already fully acclimated prior to deployment and no abnormal valve behavior or evidence of technical artefacts were detected during this period. Seasonal variations in VOA broadly paralleled the observed growth phases, with higher VOA values occurring during periods of steeper shell growth. Consistent with this pattern, a significant positive correlation was observed between VOA and shell growth (Spearman test: *p* = 0.0129). The increase in SE toward the end of the experiment reflects the reduced number of individuals following progressive technical loss of signals.

### The influence of environmental parameters on *Ostrea edulis* shell growth

Linear mixed-effects models were used to assess the influence of environmental factors on shell growth. The outputs of the Simple Trait-Environment Relationships and Multiple Trait-Environment Relationships models are summarized in Table 2. In the first model, temperature (χ² = 63) was the strongest predictor, followed by photoperiod direction (χ² = 16) and photoperiod (χ² = 12). Chlorophyll *a* (χ² = 7) and water level (χ² = 4) had minor significant effects, whereas sound, turbidity, and salinity were non-significant. The second model, which tested temperature, photoperiod, and photoperiod direction, as well as their interactions, showed that the interaction between temperature and photoperiod direction (χ² = 13) and photoperiod direction alone (χ² = 10) had the most potent effects on daily shell growth. The synergy between photoperiod and photoperiod direction (χ² = 5) and the impact of photoperiod alone (χ² = 4) are also significant, while the effects of temperature alone and in interaction with photoperiod are not significant.

**Table 2:** Results from linear mixed models examining the influence of environmental parameters on *Ostrea edulis* shell growth. Outputs from linear mixed models consider the influence of different fixed factors (photoperiod, photoperiod direction, water temperature, water level, underwater sound, chlorophyll *a* concentration, salinity, and turbidity) on the individuals’ daily shell growth over the study period (540 days) for the first model. A second model, explores the influence of the three most significant predictors from the first model (based on χ²) along with all two-way interactions on the individuals’ shell growth during the study period. Significant p-values (p < 0.05) are indicated in bold.

Growth				
Single Trait-Environment Relationships model				
Growth ~ Photop. + Photop. dir. + Temp. + Water level + Sound + Chl. *a* + Salinity + Turbidity + (1 | Ind)				
Parameters	*β*	*SE*	*χ2*	*p*
Photop.	−0.01	0.00	12	**< 0.001**
Photop. dir.	−0.04	0.01	16	**< 0.001**
Temp.	0.01	0.00	63	**< 0.001**
Water level	0.12	0.06	4	**0.047**
Sound	0.01	0.00	0	0.483
Chl. *a*	−0.01	0.00	7	**0.010**
Salinity	0.01	0.00	1	0.358
Turbidity	0	0.00	0	0.735

Multiple Trait-Environment Relationships model				
Growth ~ Photop. + Photop. dir. + Temp. + Temp.: Photop. + Temp.:Photop. dir. + Photop.:Photop. dir. + (1 | Ind)				
Photop.	−0.01	0.00	4	**0.05**
Photop. dir.	−0.07	0.02	10	**0.001**
Temp.	−0.01	0.01	2	0.13
Temp. x Photop.	0.00	0.00	3	0.07
Temp. x Photop. dir.	0.01	0.00	13	**< 0.001**
Photop. x Photop. dir.	−0.01	0.00	5	**0.02**

Photop.: photoperiod (daylength); Photop. dir.: photoperiod direction (increasing or decreasing daylength); Temp.: water temperature; Chl. a: chlorophyll a concentration. Significant values are in bold.

### Biological rhythms of *Ostrea edulis* valve behavior

**Fig. 5:**
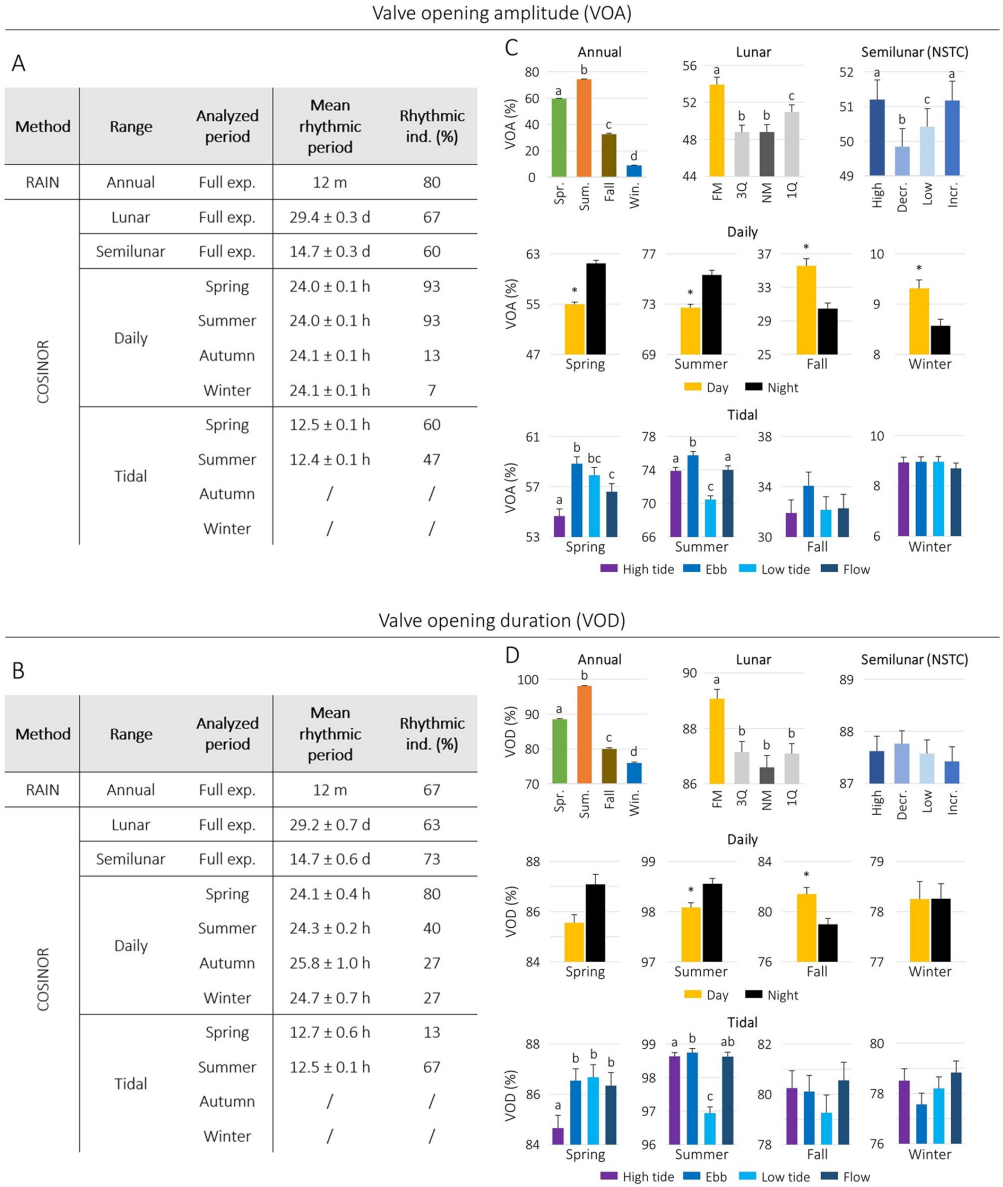
Biological rhythms of *Ostrea edulis* valve behavior. (**A**, **B**) Results of chronobiological analysis on (**A**) the individual valve amplitude dataset (VOA, %, *n* = 15) and (**B**) the individual valve opening duration dataset (VOD, %, *n* = 15), testing annual, lunar and semilunar rhythms during the field experiment, and daily and tidal rhythms during four seasons: spring (20/03/2023 to 20/06/2023), summer (21/06/2023 to 22/09/2023), autumn (23/09/2023 to 21/12/2023) and winter (22/12/2023 to 19/03/2024). (**C**, **D**) Mean VOA (**C**) and VOD (**D**) segmented according to geophysical cycles’ phases (*n* = 15). The annual histogram displays the mean VOA and VOD values by season. The lunar histogram displays the mean VOA and VOD by moon phases (FM = Full Moon, 3Q = 3rd Quarter, NM = New Moon, and 1Q = 1 st Quarter). The semilunar histogram (NSTC = neap-spring tidal cycle) displays the mean VOA and VOD across the tidal coefficient categories (high, decreasing, low, and increasing). Daily histograms display the mean VOA and VOD values during both day and night periods. Tidal histograms show the mean VOA and VOD across to the tide phase (high tide, ebb, low tide, and flow).

Chronobiological analysis revealed how *O. edulis* synchronizes its behavior with various geophysical cycles (Fig. 5). We assessed rhythmicity in VOA and VOD across annual, lunar, semilunar, daily, and tidal cycles using RAIN and Cosinor analyses (Fig. 5A,B).

For VOA (Fig. 5A,C), significant annual rhythms (12-month cycle) were observed in 80% of individuals (Fig. 5A), with the highest VOA in summer and the lowest in winter (Fig. 5C). Lunar rhythms of 29.2 ± 0.7 days, aligned with the ~ 29.5 days synodic cycle of the moon, were detected in 67% of individuals (Fig. 5A). Phase analysis showed that VOA peaked at full moon, was intermediate at first quarter moon, and lowest at new moon and third quarter moon (Fig. 5C). Semilunar rhythms appeared in 60% of individuals with an average period of 14.7 ± 0.6 days, matching the ~ 14.7 days semilunar cycle (Fig. 5A). VOA was highest during high and increasing tides (Fig. 5C). Daily rhythms, following a 24-hour cycle, were observed across all seasons. However, the proportion of individuals showing significant daily rhythms was high in spring and summer (93% in both) and markedly lower in autumn and winter (13% and 7%, respectively) (Fig. 5A). Additionally, individuals were significantly nocturnal in spring and summer, shifting to diurnal patterns in autumn and winter (Fig. 5C). Tidal rhythms, aligned with a 12.4-hour cycle, were evident in spring and summer (60% and 47% of rhythmic individuals, respectively), but absent in autumn and winter (Fig. 5A). During spring and summer, the highest activity levels coincided with ebb tides (Fig. 5C).

For VOD (Fig. 5B, D), similar patterns were observed. Annual rhythms were observed in 67% of individuals, with peak VOD in summer and minimum in winter. Lunar rhythms (29.2 ± 0.7 days) were present in 63% of individuals (Fig. 5B), with peaks around the full moon (Fig. 5D). Semilunar rhythms (14.7 ± 0.6 days) were significant in 73% of individuals, although no significant differences in VOD were observed according to the tidal coefficient partitioning applied in this study (Fig. 5B,D). Daily rhythms were observed in 80% of individuals in spring, 40% in summer, and 27% in autumn and winter (Fig. 5B). At the group level, the mean VOD activity was significantly nocturnal in summer, and shifted to diurnal in autumn, while no significant day-night differences were detected in spring and winter (Fig. 5D). Tidal rhythms were present in spring and summer (13% and 67% of rhythmic individuals, respectively), with the lowest VOD observed during high tide in spring and during low tide in summer (Fig. 5B,D). As observed for VOA, tidal rhythmicity in VOD disappeared in autumn and winter, and no differences were observed according to tidal cycle phases at these seasons (Fig. 5B,C).

### Annual rhythms of *Ostrea edulis* gene expression

**Fig. 6:**
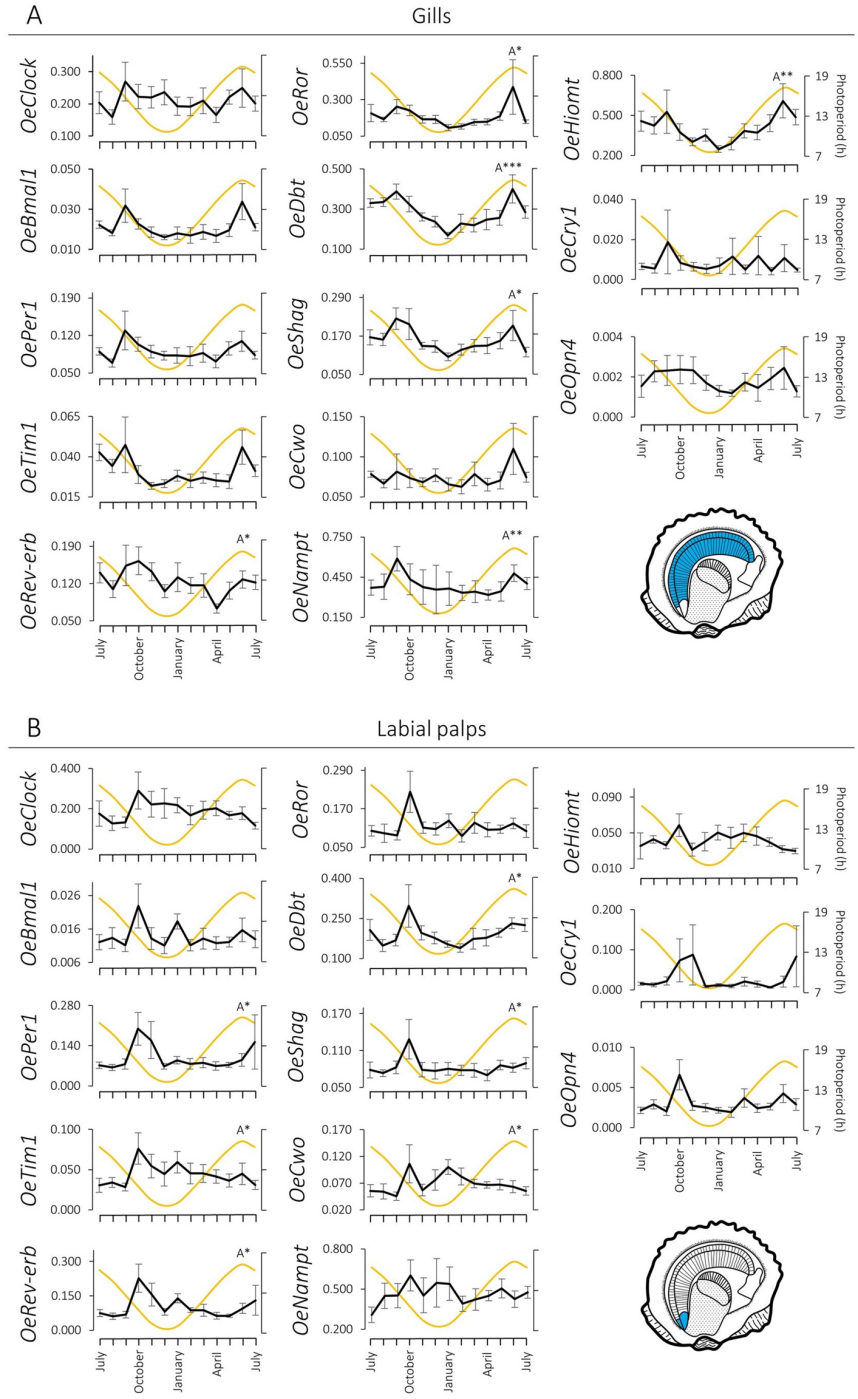
Annual profiles of expression of genes involved in the clock machinery of *O. edulis* in gills and labial palps in the field. Relative mRNA levels (mean ± SE, *n* = 8–10 oysters per time point) of nine core circadian clock and clock-related genes (*OeClock*, *OeBmal1*, *OePer1*, *OeTim1*, *OeRev-erb*, *OeRor*, *OeDbt*, *OeShag*, *OeCwo*), a clock-controlled metabolic gene (*OeNampt*), a gene involved in melatonin synthesis (*OeHiomt*), and two photoreceptor genes (*OeCry1*, *OeOpn4*) in (**A**) gill tissues and (**B**) labial palps during 13 months of the field experiment (monthly sampling around solar noon, July 2023 to July 2024). Photoperiod is shown in yellow. Statistically significant annual rhythms (RAIN algorithm) are noted with asterisks: A* = *p* < 0.05, A** = *p* < 0.01, A*** = *p* < 0.001. Oyster gills and labial palps are colored in blue in the diagrams (oyster diagrams: ©2020, Native Oyster Network - UK & Ireland, Native Oyster Restoration Alliance).

Annual expression profiles and RAIN analyses of 13 genes involved in circadian clock function (*OeClock*, *OeBmal1*, *OePer*, *OeTim1*, *OeRev-erb*, *OeRor*, *OeDbt*, *OeShag*, *OeCwo*), metabolic regulation (*OeNampt*), melatonin synthesis (*OeHiomt*), and photoreception (*OeCry1*, *OeOpn4*) were assessed in gills and labial palps sampled monthly from July 2023 to July 2024 (Fig. 6).

Overall, expression patterns in gill tissue (Fig. 6A) showed two annual peaks, one in September 2023 and the other in June 2024. This is clearly noticeable for genes such as *OeBmal1*, *OeTim1*, *OeDbt*, *OeShag*, and *OeHiomt*. Other genes, such as *OePer1* and *OeCry1* showed a single peak in September, while *OeRor* and *OeCwo* peaked in June. The winter months were associated with lower expression levels for several genes, including *OeBmal1*, *OeTim1*, *OeDbt*, *OeShag*, and *OeHiomt*, suggesting seasonal downregulation. Six genes exhibited significant annual rhythmicity: *OeRev-erb*, *OeRor*, *OeDbt*, *OeShag*, *OeNampt*, and *OeHiomt*.

In labial palps (Fig. 6B), the annual patterns differ from those observed in gills, highlighting tissue-specific regulation. Notably, most genes showed a peak of expression in October 2023, one month later than in gills. Here, six genes were identified as rhythmic: *OePer1*, *OeTim1*, *OeRev-erb*, *OeDbt*, *OeShag*, and *OeCwo*.

## Discussion

This study provides a first comprehensive, long-term analysis of *Ostrea edulis* phenology in its natural environment, based on in situ monitoring of valve activity and shell growth over 540 days, combined with monthly gene expression profiling. Our results show that *O. edulis* valve activity exhibits a complex interaction of biological rhythms, including diel (~ 24 h), tidal (~ 12.4 h), semilunar (~ 14.8 d), lunar (~ 29.5 d), and annual (~ 365 d) cycles. The annual rhythm was reflected in both behavior and growth, with peaks in valve activity and shell growth during spring and summer, and a marked reduction in winter entering a resting state. Mixed models revealed that the interaction between photoperiod and temperature was the primary driver of seasonal variation in activity and growth. Additional environmental cues, such as chlorophyll *a* and underwater noise, had secondary but notable effects. At the molecular level, several circadian clock and clock-related genes displayed significant annual oscillations in both gills and labial palps, suggesting the presence of internal timekeeping mechanisms.

### Investigation of *Ostrea edulis* phenology in its natural environment

#### Seasonal patterns in annual behavioral, growth and molecular rhythms in *O. edulis*

Annual rhythms are essential in aligning physiology and behavior with seasonal environmental changes, thereby improving survival and reproductive success^79^. Our study showed that the phenology of *O. edulis* clearly exhibited annual rhythms at the molecular, behavioral, and physiological levels. In this study, we use the term phenology in a broad sense to describe the seasonal organization of behavioral, physiological, and molecular processes in relation to recurring environmental cycles. Accordingly, valve activity, shell growth, and many gene expressions related to the circadian clock machinery and clock outputs peaked during spring and summer and decreased significantly during autumn and winter. A pronounced winter slowdown is a common strategy among marine organisms. For instance, crustaceans such as copepods^54^ remain inactive for several months during autumn and winter to cope with unfavorable conditions. Bivalves also reduce activity during short-day seasons, but *O. edulis* in Helgoland displayed a much stronger winter slowdown than *Crassostrea gigas* at southern latitudes^43^. However, comparable damped activities have been observed in the mussel *Mytilus spp.* in the Arctic during harsh seasons^41^^,64^. It can be hypothesized that the pronounced winter slowdown of *O. edulis* observed in this study would be less significant at lower latitudes. Testing this hypothesis will require comparative studies, both across bivalve species at similar latitudes and within *O. edulis* populations along its distribution range^36^. Controlled laboratory experiments manipulating temperature under constant photoperiod conditions could also help disentangle the respective effects of temperature and seasonal light cues. Our findings also showed that annual rhythms in *O. edulis* were synchronized with environmental factors, photoperiod-temperature interaction being the strongest predictor of valve activity and shell growth. Photoperiod is the most stable and predictable cue for annual environmental cycles, indirectly shaping other factors such as temperature and food availability^80^. Consequently, it is the most reliable time-giver for endogenous circannual clocks, allowing anticipation and synchronization with predictable annual environmental cycles^80^^,81^. In *O. edulis*, it could allow physiology to be adjusted at each time of the year, anticipating energy conservation during cold or food-poor periods and anticipating increased activity in spring and summer to optimize reserve accumulation and gametogenesis. While the photoperiod-synchronized circannual clock would control the annual timing of biological processes, most of physiological processes depend on the ambient temperature since oysters are poikilotherms, emphasing the role of photoperiod-temperature interaction on *O. edulis* behavior and growth observed in this study.

Recent investigations have identified key circadian clock and clock-related genes in *O. edulis*^45^, providing molecular evidence for temporal regulation. The analyzed clock and clock-related genes, are increasingly recognized for their role in longer-term timekeeping^81^^,82^. Several core and clock-related genes, such as *OePer1*, *OeTim1*, *OeRev-erb*, *OeRor*, *OeDbt*, *OeShag*, *OeCwo*, *OeHiomt*, and *OeNampt*, exhibited annual rhythmicity in the gills and labial palps of *O. edulis*, suggesting the possible existence of an endogenous circannual timing system involving the circadian clock machinery. Notably, *OeHiomt*, involved in melatonin synthesis, showed an annual rhythm of expression in *O.edulis*. Given melatonin’s known role in seasonal timing and reproductive regulation in animals^55^^,83^^,84^, its expression pattern in *O. edulis* suggests a role in photoperiod-driven synchronization of physiology, including seasonal behaviors and physiological functions.

Overall, the alignment of behavioral, physiological, and molecular rhythms, combined with the absence of observed mortality, reflects strong synchronization with environmental cycles and supports that *O. edulis* was healthy and well adapted to its habitat during the experiment in Helgoland.

#### Daily rhythm in valve activity

Our results showed that *O. edulis* exhibits daily rhythms of valve behavior. The behavioral and molecular daily rhythms of *O. edulis* have been previously observed in laboratory studies under controlled conditions (Le Moal et al. 2025; *under review*), and were found to be maintained under constant light, demonstrating the presence of a functional endogenous circadian clock. These results supported the fact that the daily rhythms observed in the field reflect the output of an internal anticipation mechanism shaped by evolution and synchronized by daily light-dark cycles, rather than a direct response to environmental signals^40^. Interestingly, our study showed that *O. edulis’* daily rhythms of valve activity were stronger in spring and summer, and weakened in autumn and winter. Such annual modulations of daily rhythms were previously observed in the oyster *C. gigas* or the noble pen shell *Pinna nobilis*, and suggest a circannual control of the circadian clock^43^^,85^^,86^. Moreover, diel activity phase in *O. edulis* shifted with the time of year: oysters are nocturnal during long-day seasons and switch to diurnal activity during short-day seasons. Such seasonal phase reversal has also been reported in the oyster *C. gigas*^85^, as well as in the burbot *Lota lota*^87^^,88^. However, in *C. gigas*, the opposite trend occurs, suggesting possible temporal niche partitioning between these cohabiting species^89^^–91^. This daily rhythm flexibility may optimize feeding, energy use, or predator avoidance across seasons.

#### Rhythms related to lunar cycles

In addition to daily and annual cycles, our study revealed lunar (~ 29.5 d), semilunar (~ 14.8 d), and tidal (~ 12.4 h) rhythmicities. These cycles are well-established in marine environments, often associated with either light variation (from moon phases) or mechanical forces (from tidal changes)^42^^,92^.

In our study, we clearly identified a lunar rhythm (29.5 days) in *O. edulis* valve activity, with peak activity around full moon phases. Similar moonlight-related rhythms occur in bivalves such as *C. gigas*^43^^,93^, *Mytilus spp.*^63^^,64^, *P. nobilis*^94^, and in many other marine invertebrates such as the annelid *Platynereis dumerilii*^37^^,95^. These results support the idea that *O. edulis* detected low-intensity moonlight, possibly via specialized photoreceptors^96^. Although the mechanisms underlying moonlight perception remain unresolved, photoreceptive molecules such as cryptochromes and opsins have been proposed as potential candidates^97^^,98^, and orthologs of these genes were identified in our previous characterization of the molecular clock and photoreceptive pathways *O. edulis*^45^. Further experiments would be needed to decipher whether the observed lunar rhythm in *O. edulis* is a direct response to moonlight or the result of a synchronized endogenous circalunar clock^37^.

In contrast, the semilunar rhythm (14.8 days) and tidal rhythm (12.4 h) observed in *O. edulis* are more likely linked to mechanical forces, such as tidal amplitude and water pressure changes. First, a tidal rhythm (~ 12.4 h) was also observed in *O. edulis*, although it was expressed by fewer individuals compared to diel rhythms, and only during spring and summer. This pattern differs from what is commonly observed in other bivalves where tidal rhythms are more consistently and widely expressed across individuals and seasons, such as observed in *C. gigas*^42^^,43^^,65^ or the intertidal clams *Chione stutchburyi*^99^ and *Austrovenus stutchburyi*^100^. The rarity of tidal rhythms in *O. edulis* in our study could be attributed to the relatively low tidal amplitude (< 2 m) at our subtidal study site, which may reduce the strength of tidal cues (pressure and current variations) that may be too subtle to entrain tidal rhythms in all organisms. Nevertheless, the seasonal expression of both tidal and diel rhythms suggests the presence of a plastic clock mechanism, enabling organisms to anticipate predictable environmental fluctuations and optimize their physiological processes accordingly^101^. This interaction between tidal and diel rhythms has been described in other marine invertebrates, such as *C. gigas* bivalves^43^^,65^^,103^ or crustaceans like the isopod *Excirolana chiltoni*^104^ or the copepod *Calanus finmarchicus*^102^.

Additionally, the semilunar rhythm reflects the neap-spring tidal cycle, a biweekly mechanical cue that modulates tidal amplitude and thus reinforces the expression of tidal rhythmicity. This semilunar cycle was observed in our study through higher valve amplitude during spring tides (high tidal coefficient) and aligns with findings in other bivalves such as *C. gigas*^42^^,43^ and *P. nobilis*^94^ or in the isopod *Eurydice pulchra*^105^. To fully understand the endogenous basis of these rhythms, both laboratory and field studies of clock gene expression across tidal cycles are needed to determine whether *O. edulis* tidal rhythms are endogenously generated or purely reactive to environmental cues.

### Environmental drivers and emerging stressors

Mixed-effects models revealed that the interaction between photoperiod and temperature was the strongest predictor of annual rhythms in *O. edulis*. Our findings also suggest contributions from additional environmental cues: underwater sound significantly affected valve opening amplitude, while chlorophyll *a* influenced valve opening duration, likely reflecting adjustments in feeding behavior. Turbidity and salinity, though less influential, showed notable effects as already showed by Bamber^106^ for the salinity. Water level, reflecting tidal cycles, had a minor but noticeable effect despite low tidal amplitude at our subtidal site.

This dependency on environmental synchronization also introduces vulnerability. Firstly, Artificial light at night (ALAN) disrupts natural photic cycles, potentially desynchronizing circadian and seasonal rhythms by interfering with photoperiod perception^25,107–110^. Among the surfaces exposed to ALAN, coastal areas are strongly impacted^108^. Concerning *O. edulis’* habitats, i.e. Europe’s coastline areas, 54% are estimated to be light-polluted, with sky brightness increasing by 9.6% per year^111^^,112^. Such shifts in light regimes could impair internal clock entrainment in oysters, as observed in *C. gigas*^109,113^^,114^, and have already been shown to disrupt *O. edulis* daily rhythm^25^, potentially leading to altered physiological functions under clock control such as behavior, growth or reproduction^65^. Secondly, climate change could also threaten the fitness of *O. edulis* by decoupling temperature from photoperiod, causing phenological mismatches during critical stages like gametogenesis and larval development^115^^,116^. Also, noise pollution is an emerging anthropogenic stressor that could therefore disrupt natural behavior rhythms^117^^–119^. In this study we observed that low frequency underwater sound had a significant influence on valve activity in *O. edulis*, even under conditions of relatively low ambient sound intensity and variation at our site. This result corroborates previous studies showing behavioral responses of bivalves species such as *C. gigas* and *M. edulis* to low-frequency vibrations (< 1000 Hz)^48^^,120^. Beyond direct behavioral responses, sound may serve as a mechanical *zeitgeber* for tidal rhythms^121^. However, while the long-term effects of acoustic disturbance on *O. edulis* remain to be deciphered, our findings suggest that noise exposure could chronically disrupt their behavior, potentially impacting individual fitness and population dynamics^117^^,118^. Finally, anthropogenic activities can alter salinity through precipitation changes, freshwater runoff, or wastewater discharge^122^, and increase turbidity through dredging and bottom trawling^123^ or through sediment extraction or discard. In our study, we showed that both salinity and turbidity had significant effects on valve behavior in *O. edulis*, suggesting that shifts in salinity could disrupt osmoregulation^60^^,61^, while increased turbidity may impair gill function and feeding efficiency^62^. This highlights the sensitivity of oyster to environmental degradation and points to potential risks for population stability under increasing anthropogenic pressures.

### Conservation and management considerations

These findings have important implications for conservation and restoration strategies targeting *O. edulis* reefs. The demonstration of both environmental synchronization in the field and endogenous rhythmicity indicates that *O. edulis* is evolutionarily adapted to its habitat, supporting the potential for sustainable oyster reef habitat restoration. However, the reliance on environmental cues also highlights vulnerability to anthropogenic stressors. Restoration planning should therefore incorporate rhythmic biology into site selection and timing. Locations exposed to artificial light, disrupted tidal regimes, acoustic disturbance, elevated turbidity or thermal instability may hinder proper rhythm entrainment, potentially reducing fitness and increasing vulnerability to stressors, including pathogens like *Bonamia ostreae*, a key driver of population collapse^124^^,125^. More broadly, the rhythmic parameters identified in this study, such as valve activity patterns, shell growth dynamics, and their synchronization with environmental cycles, could serve as practical indicators for assessing habitat suitability and physiological condition in oyster farming and restoration sites. Monitoring the stability or disruption of these rhythms may help identify environments favorable for oyster performance and long-term resilience.

## Conclusion

This study reveals that *O. edulis* exhibits multiple synchronized biological rhythms, including daily, tidal, lunar, semilunar, and annual cycles, across molecular, behavioral, and physiological levels in its natural environment. Valve behavior and growth are mainly driven by photoperiod and temperature, two key environmental cues, with additional modulation by water level, chlorophyll *a* concentration (used here as a proxy for phytoplankton density), ambient sound, turbitidy and salinity. The alignment between internal clocks and environmental cycles, along with the absence of mortality over 540 days, suggests that the oysters were well synchronized and healthy. While this synchronization with various environmental factors enhances ecological adaptability, it also raises vulnerability to anthropogenic stressors like light, noise, and pollution. Understanding *O. edulis* rhythms offers valuable insights for monitoring health, resilience, and habitat suitability, supporting adaptive conservation and restoration in a rapidly changing coastal environment. Future research should focus on assessing the resilience of these rhythms under disturbed conditions to better support conservation and restoration of *O. edulis* into changing coastal ecosystems.

## Supplementary Information

Below is the link to the electronic supplementary material.

Supplementary Material 1

## Data Availability

The datasets used and analysed during the current study (individual hourly valve behavior data (VOA and VOD), temperature and underwater noise recordings) are publicly available in PANGAEA^®^ - Data Publisher for Earth & Environmental Science (https://doi.pangaea.de/10.1594/PANGAEA.983652).
