# Supplementary material for: The phenology of the European flat oyster (Ostrea edulis) and its environmental drivers
Source: Sci Rep. 2026 Jul 27;16:23359. doi: 10.1038/s41598-026-63105-x (PMC13408198; doi:10.1038/s41598-026-63105-x)
Supplement: Supplementary file 1 — Supplementary Material 1 [file 41598_2026_63105_MOESM1_ESM.docx]

Supplementary Information

**The phenology of the European flat oyster (*Ostrea edulis*) and its environmental drivers**

Alexandre LE MOAL, Damien TRAN, Laura PAYTON, Yannick GEEREBAERT, Bernadette POGODA, Bettina MEYER

Journal: Scientific reports

Corresponding author: Alexandre Le Moal / Bettina Meyer

E-mail: [alexandre.lemoal@awi.de](mailto:alexandre.lemoal@awi.de) / bettina.meyer@awi.de

**The following items are provided:**

**Table S1:** Individual biometric data of oysters used in the behavioral experiment.

**Table S2:** Individual biometric data of oysters used in the molecular experiment.

**Figure S1:** Mean daily valve opening amplitude (VOA, %) and valve opening duration (VOD, %) of *Ostrea edulis* at the individual level during our experiment on Helgoland.

**Figure S2.** Visual representation of daily and tidal patterns in oyster valve activity.

**Table S3:** Accession number of identified genes of interest in *Ostrea edulis*.

**Table S1. Individual biometric data of oysters used in the behavioral experiment.** Shell length and shell width measurements (mm) of the 16 *Ostrea edulis* individuals monitored continuously by HFNI valvometry throughout the study. Individual n°7 died a few days after deployment and was therefore excluded from the analyses.

| **Oyster n°** | **Length (mm)** | **Width (mm)** |
| --- | --- | --- |
| 1 | 70 | 86 |
| 2 | 79 | 84 |
| 3 | 82 | 86 |
| 4 | 81 | 84 |
| 5 | 73 | 78 |
| 6 | 69 | 75 |
| 7 | 80 | 82 |
| 8 | 84 | 76 |
| 9 | 74 | 76 |
| 10 | 76 | 78 |
| 11 | 75 | 73 |
| 12 | 74 | 78 |
| 13 | 82 | 76 |
| 14 | 76 | 83 |
| 15 | 74 | 69 |
| 16 | 82 | 82 |

**Table S2. Individual biometric data of oysters used in the molecular experiment.** Shell length and shell width measurements (mm) of the 134 *Ostrea edulis* individuals collected during monthly sampling for gene expression analyses. Months indicate experimental sampling time points, with the exact sampling date provided in parentheses. Sampling dates correspond to standardized monthly sampling events scheduled according to the lunar phase.

| **Oyster n°** | **Length (mm)** | **Width (mm)** |
| --- | --- | --- |
| **July 2023** (sampling date: 10 July 2023) | | |
| 1.1 | 75 | 75 |
| 1.2 | 70 | 75 |
| 1.3 | 70 | 68 |
| 1.4 | 75 | 65 |
| 1.5 | 75 | 80 |
| 1.6 | 70 | 65 |
| 1.7 | 90 | 80 |
| 1.8 | 75 | 70 |
| **August 2023** (sampling date: 11 August 2023) | | |
| 2.1 | 80 | 66 |
| 2.2 | 90 | 90 |
| 2.3 | 70 | 80 |
| 2.4 | 80 | 80 |
| 2.5 | 85 | 80 |
| 2.6 | 75 | 80 |
| 2.7 | 80 | 70 |
| 2.8 | 78 | 75 |
| 2.9 | 90 | 90 |
| 2.10 | 85 | 105 |
| **September 2023** (sampling date: 8 September 2023) | | |
| 3.1 | 95 | 95 |
| 3.2 | 105 | 90 |
| 3.3 | 85 | 80 |
| 3.4 | 95 | 90 |
| 3.5 | 80 | 78 |
| 3.6 | 90 | 90 |
| 3.7 | 85 | 85 |
| 3.8 | 100 | 80 |
| 3.9 | 90 | 98 |
| 3.10 | 95 | 85 |
| **October 2023** (sampling date: 6 October 2023) | | |
| 4.1 | 83 | 79 |
| 4.2 | 95 | 90 |
| 4.3 | 90 | 77 |
| 4.4 | 89 | 89 |
| 4.5 | 80 | 92 |
| 4.6 | 85 | 65 |
| 4.7 | 80 | 82 |
| 4.8 | 90 | 90 |
| 4.9 | 100 | 84 |
| 4.10 | 79 | 90 |
| **November 2023** (sampling date: 7 November 2023) | | |
| 5.1 | 85 | 85 |
| 5.2 | 85 | 85 |
| 5.3 | 95 | 90 |
| 5.4 | 90 | 80 |
| 5.5 | 85 | 75 |
| 5.6 | 95 | 80 |
| 5.7 | 85 | 80 |
| 5.8 | 85 | 60 |
| 5.9 | 90 | 90 |
| 5.10 | 90 | 90 |
| 5.11 | 95 | 95 |
| **December 2023** (sampling date: 6 December 2023) | | |
| 6.1 | 83 | 90 |
| 6.2 | 80 | 75 |
| 6.3 | 95 | 100 |
| 6.4 | 97 | 90 |
| 6.5 | 85 | 42 |
| 6.6 | 95 | 85 |
| 6.7 | 100 | 90 |
| 6.8 | 85 | 75 |
| 6.9 | 80 | 65 |
| 6.10 | 80 | 75 |
| 6.11 | 85 | 85 |
| **January 2024** (sampling date: 17 January 2024) | | |
| 7.1 | 80 | 67 |
| 7.2 | 85 | 75 |
| 7.3 | 85 | 75 |
| 7.4 | 100 | 80 |
| 7.5 | 77 | 75 |
| 7.6 | 85 | 80 |
| 7.7 | 80 | 75 |
| 7.8 | 80 | 80 |
| 7.9 | 75 | 67 |
| 7.10 | 75 | 75 |
| **February 2024** (sampling date: 12 February 2024) | | |
| 8.1 | 80 | 70 |
| 8.2 | 90 | 75 |
| 8.3 | 80 | 70 |
| 8.4 | 80 | 85 |
| 8.5 | 85 | 75 |
| 8.6 | 85 | 85 |
| 8.7 | 95 | 95 |
| 8.8 | 95 | 80 |
| 8.9 | 90 | 75 |
| 8.10 | 90 | 85 |
| **March 2024** (sampling date: 6 March 2024) | | |
| 9.1 | 75 | 70 |
| 9.2 | 80 | 65 |
| 9.3 | 70 | 80 |
| 9.4 | 85 | 75 |
| 9.5 | 90 | 75 |
| 9.6 | 95 | 75 |
| 9.7 | 80 | 70 |
| 9.8 | 70 | 65 |
| 9.9 | 85 | 65 |
| 9.10 | 85 | 75 |
| **April 2024** (sampling date: 3 April 2024) | | |
| 10.1 | 70 | 72 |
| 10.2 | 82 | 75 |
| 10.3 | 80 | 70 |
| 10.4 | 75 | 65 |
| 10.5 | 75 | 70 |
| 10.6 | 80 | 75 |
| 10.7 | 95 | 70 |
| 10.8 | 90 | 80 |
| 10.9 | 90 | 100 |
| 10.10 | 85 | 80 |
| **May 2024** (sampling date: 29 April 2024) | | |
| 11.1 | 85 | 85 |
| 11.2 | 100 | 95 |
| 11.3 | 95 | 80 |
| 11.4 | 85 | 85 |
| 11.5 | 80 | 85 |
| 11.6 | 80 | 75 |
| 11.7 | 85 | 75 |
| 11.8 | 80 | 75 |
| 11.9 | 80 | 85 |
| 11.10 | 90 | 85 |
| 11.11 | 80 | 85 |
| **June 2024** (sampling date: 30 May 2024) | | |
| 12.1 | 90 | 85 |
| 12.2 | 90 | 95 |
| 12.3 | 90 | 90 |
| 12.4 | 80 | 80 |
| 12.5 | 85 | 80 |
| 12.6 | 90 | 85 |
| 12.7 | 90 | 80 |
| 12.8 | 105 | 80 |
| 12.9 | 95 | 85 |
| 12.10 | 70 | 80 |
| 12.11 | 85 | 80 |
| **July 2024** (sampling date: 27 June 2024) | | |
| 13.1 | 85 | 80 |
| 13.2 | 80 | 80 |
| 13.3 | 85 | 70 |
| 13.4 | 80 | 80 |
| 13.5 | 80 | 80 |
| 13.6 | 95 | 95 |
| 13.7 | 90 | 80 |
| 13.8 | 90 | 70 |
| 13.9 | 80 | 75 |
| 13.10 | 85 | 80 |
| 13.11 | 100 | 90 |
| 13.12 | 100 | 80 |

**
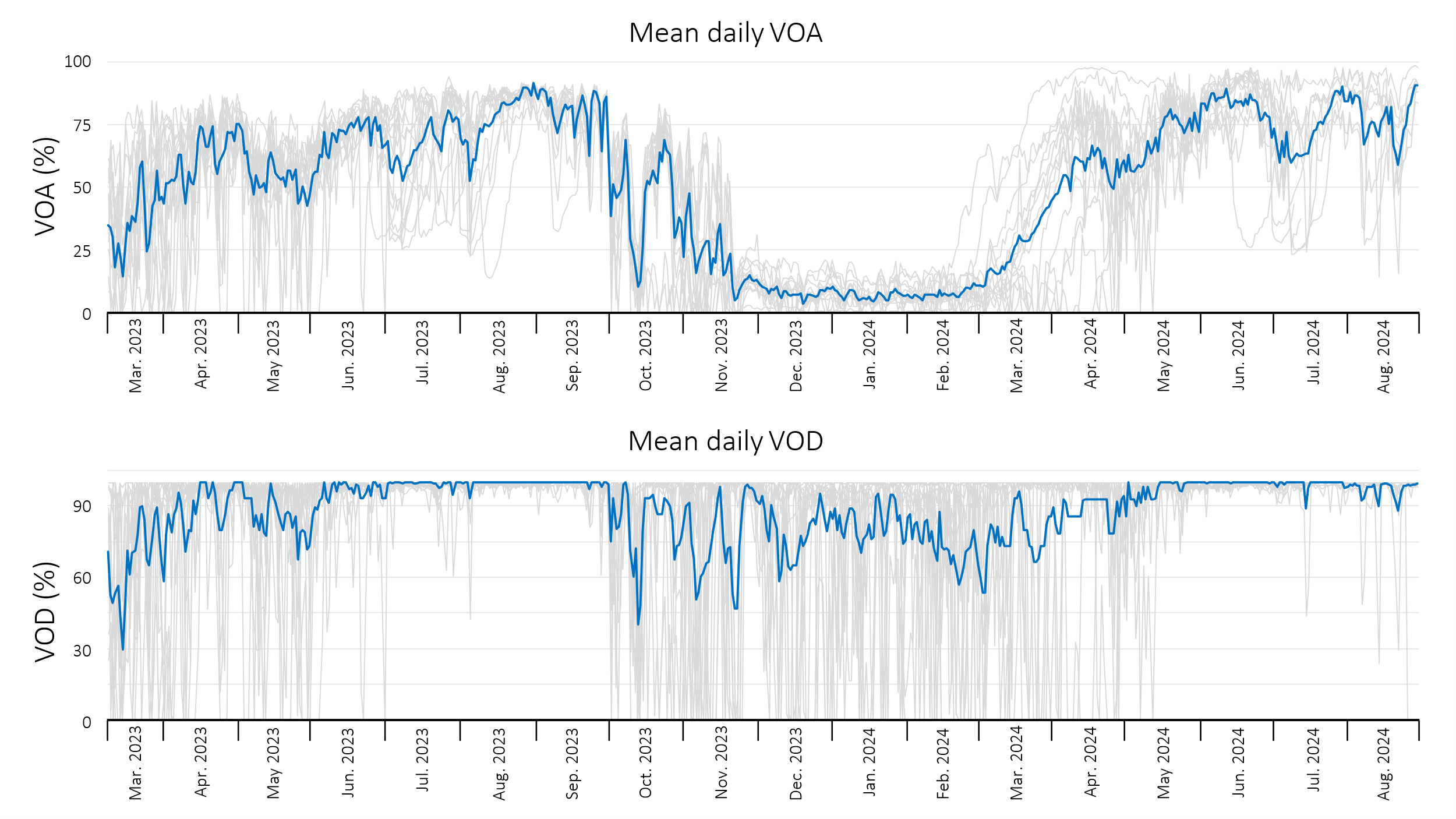
**

**Figure S1.** **Mean daily valve opening amplitude (VOA, %) and valve opening duration (VOD, %) of *Ostrea edulis* at the individual level during our experiment on Helgoland.** Grey lines represent the valve activity for each individual. The blue bold lines represent the daily mean.

**
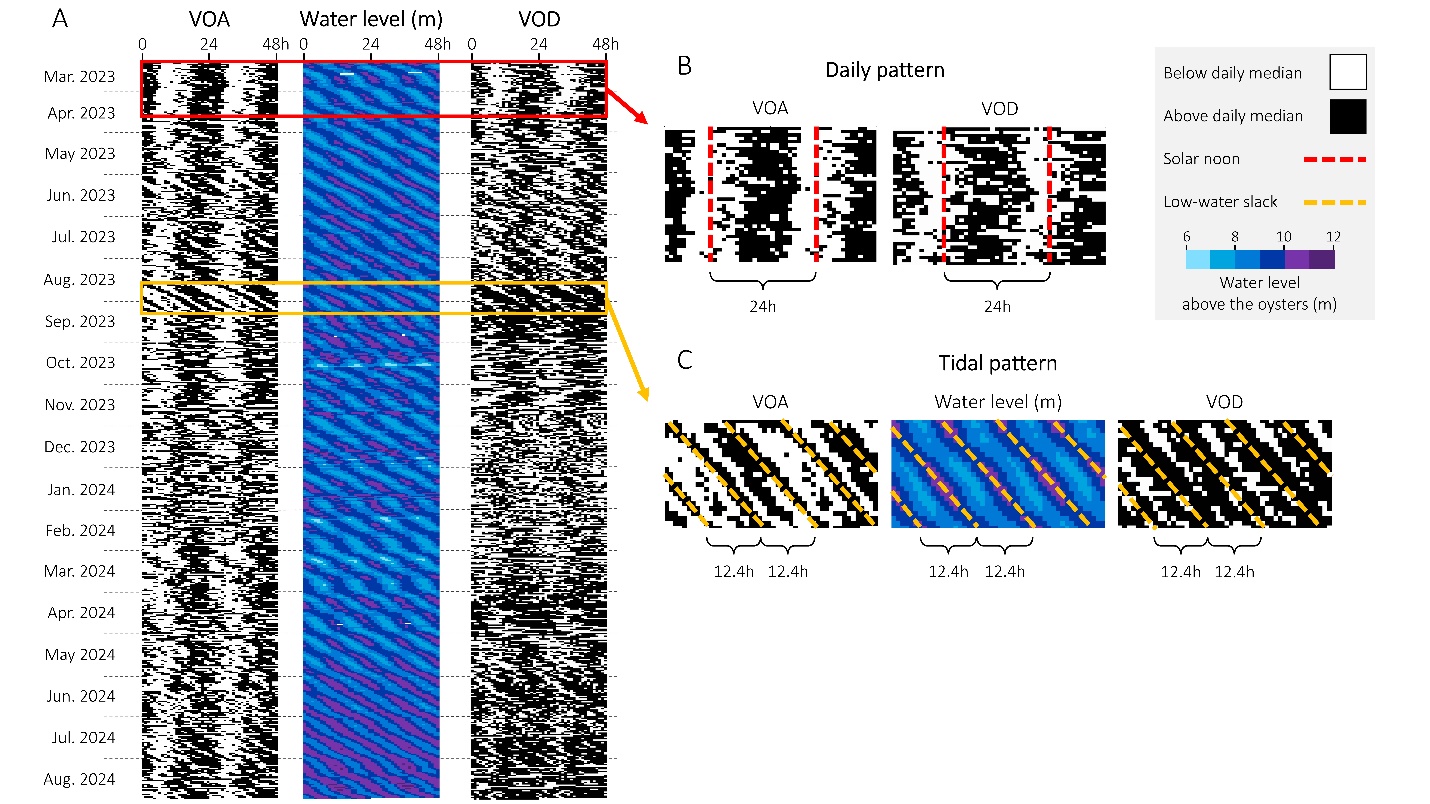
**

**Figure S2. Visual representation of daily and tidal patterns in oyster valve activity.** (A) Actograms of hourly mean valve opening amplitude (VOA, %) and valve opening duration (VOD, %) shown alongside the heatmap of the hourly water level above the oysters (m). (B) Representative example of the daily valve activity pattern showing the predominant 24h rhythmicity in VOA and VOD over several consecutive days. Red dashed lines indicate solar noon. (C) Representative example of the tidal valve activity pattern showing the predominant ~12.4h rhythmicity in VOA and VOD relative to water level over several consecutive days. Orange dashed lines indicate low-water slack.

**Table S3.** **Accession number of identified genes of interest in *Ostrea edulis*.** Abbreviation and accession number for house-keeping genes used for the molecular analysis are in blue.

| **Designation** | **Abbreviation** | **Size (aa)** | **Accession Number (NCBI)** | **Forward sequence for primer design** | **Reverse sequence for primer design** |
| --- | --- | --- | --- | --- | --- |
| **Core circadian clock genes** | | | | | |
| *OeClock* | *OeClock* | 688 | XM_048900854.1 | CAGCCACCAGTACCCAGAAT | TCTGGCTCGGAGATTTCTGT |
| *OeBmal1* | *OeBmal1* | 546 | XM_048884855.1 | ACGAGAGGGTGCAGCTATGT | AGGATGTTCCGCACTGATTC |
| *OePeriod* | *OePer1* | 1514 | XM_048884858.1 | GGCACATGCTGAACAAGAGA | AGAGGAAATGGTGGCACAAG |
| *OeTimeless* | *OeTim1* | 949 | XM_048891525.1 | AGGAACCAGTGGCACGATAC | TGGGAAACACAAGACCAACA |
| **Clock-related genes** | | | | | |
| *OeRev-erb* | *OeRev-erb* | 1089 | XM_048918855.1 | CTGTTCCGCCACAATTCTTT | CCTTGGACGCAATTGAGAAT |
| *OeRor* | *OeRor* | 502 | XM_048910521.1 | CTCCCCAAGTCACCTCTCAG | GAGGGGTCATGCTGGATTTA |
| *OeDoubletime* | *OeDbt* | 426 | XM_048904586.1 | GAATCGCTGGGATACGTGTT | CAGGGATCGGCAAAAGTTTA |
| *OeShaggy* | *OeShag* | 412 | XM_048925210.1 | AGATCCAAACACCAGGTTGC | GATTGGTGTCTCCCGACAGT |
| *OeClockWorkOrange* | *OeCwo* | 586 | XM_048885548.1 | GCCCAAAGAACATTCAAGGA | GGTGCTTCTTCTGGAGCTTG |
| **Clock-controlled metabolic gene** | | | | | |
| *OeNampt* | *OeNampt* | 468 | XM_048882855.1 | ACCCAAAATGCTTCTGGTTG | TGTTGAACCACGAAATCCAA |
| **Gene involved in melatonin synthesis** | | | | | |
| *OeHiomt* | *OeHiomt* | 410 | XM_048888998.1 | TTGCAGAAGGATTGCAACAG | ATGATCATGGCATCGACAGA |
| **Photoreceptor genes** | | | | | |
| *OeCryptochrome1* | *OeCry1* | 545 | XM_048882495.1 | GTCTCGCACACTCTGTGGAA | GTCCGTGAAGTCTGGGTCAT |
| *OeMelanopsin* | *OeOpn4* | 584 | XM_048908555.1 | GACGAGGCTGGACAGAAAAC | GACGGTCCTGGTTGAGTGTT |
| **Housekeeping genes** | | | | | |
| *OeGAPDH* | *OeGapdh* | 1518 | XM_048908150.1 | TGCAATCAATGATCCCTTCA | AGCCTTTCCGCTGATCACTA |
| *OeEF1* | *OeEf1* | 1855 | XM_048885495.1 | CCAGGCCACAGAGATTTCAT | TTGACTCCGACAATGAGCTG |
| *Oe28S* | *Oe28S* | 640 | XM_048885299.1 | TGGGGAAAGTGATAGACGTTG | AACCTCCTCCTTCCCAGTGT |
